# A Comprehensive Review of Carbon Capture, Storage, and Reduction Strategies Within the Built Environment

**DOI:** 10.3390/ma18245646

**Published:** 2025-12-16

**Authors:** Eyad Abdelsalam Elsayed Hamed, Shoukat Alim Khan, Arslan Yousaf, Muammer Koç

**Affiliations:** Division of Sustainable Development, College of Science and Engineering, Hamad Bin Khalifa University, Doha P.O. Box 5825, Qatar

**Keywords:** carbon capture, carbon storage, carbon reduction, CO_2_ sequestration, sustainable construction materials

## Abstract

The built environment (BE) encompasses an enormous volume and substantial material mass. However, structures within it typically serve single, limited functions. Enhancing these structures with multifunctional capabilities holds significant potential for achieving broader sustainability goals and creating impactful environmental benefits. Among these potential multifunctional applications, carbon capture, reduction, and storage are especially critical, given the current built environment’s substantial contribution of approximately 40% of global energy and CO_2_ emissions. Keeping this potential in view, this comprehensive review critically evaluates carbon management strategies for the built environment via three interrelated approaches: carbon capture (via photosynthesis, passive concrete carbonation, and microbial biomineralization), carbon storage (employing carbonation curing, mineral carbonation, and valorization of construction and demolition waste), and carbon reduction (integrating industrial waste, alternative binders, and bio-based materials). The review also evaluates the potential of novel direct air-capture materials, assessing their feasibility for integration into construction processes and existing infrastructure. Key findings highlight significant advancements, quantify CO_2_ absorption potentials across various construction materials, and reveal critical knowledge gaps, thereby providing a strategic roadmap for future research direction toward a low-carbon, climate-resilient built environment.

## 1. Introduction

### 1.1. Background

**Figure 1:**
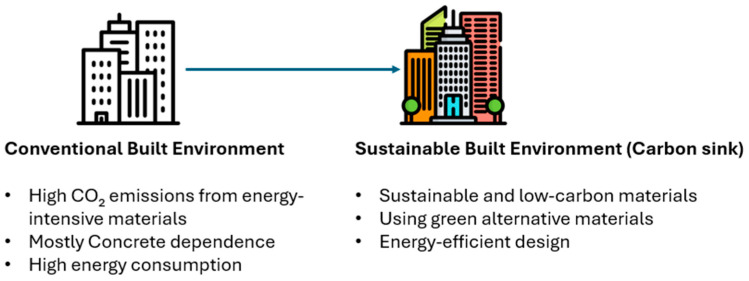
The difference between the current BE and the sustainable BE.

Climate change has become a significant topic of discussion in the 21st century, as it is linked to many types of extreme weather and natural disasters. The report by the Intergovernmental Panel on Climate Change (IPCC) stated that the temperature of the Earth had been raised by about 1.0 °C in comparison to pre-industrial levels, with suggestions that if the current trend persists, an increase of 0.2 °C per decade is assured to occur [1]. This warming has shifted extreme weather events, including rising sea levels and decreased biodiversity. There has been an estimated increase of 48% in emissions of greenhouse gases, especially carbon dioxide (CO_2_), since 1750, primarily due to human activities [2,3]. The built environment (BE) comprises all human-created surroundings, such as the buildings and infrastructure (Figure 1) has a significant bearing when it comes to the issue of climate change.

BE accounts for almost 40% of global energy and process-related CO_2_ emissions [4,5]. Ordinary Portland cement (OPC) is among the major contributors, which emits nearly 1 ton of CO_2_ per ton produced [6]. 25 billion tons of concrete are consumed annually worldwide, making it the second most often utilized construction material today, behind water [7]. This situation brings to the forefront the importance of finding ways to enhance the environmental sustainability of the construction sector. There is a growing movement to turn the built environment into a carbon sink [8]. Over 1000 cities and 130 countries have committed to achieving net-zero emissions by 2050 [9,10,11], which means that the built environment would capture more carbon than it emits, creating a positive impact on our planet. Carbon capture and storage (CCS) is a process that captures CO_2_ emissions from significant sources such as power plants, natural gas processing facilities, and industrial operations.

There are three primary techniques for CO_2_ removal: post-combustion, where CO_2_ is separated from flue gases after fuel is burned; pre-combustion, which captures CO_2_ before combustion by converting fuel into hydrogen and CO_2_; and oxy-fuel combustion, which burns fuel in pure oxygen, producing a CO_2_-rich exhaust that is easier to capture [12]. Once captured, CO_2_ is transported and stored underground in depleted oil and gas reservoirs, deep saline aquifers, or coal seams to prevent its release into the atmosphere. However, long-term storage in deep rock formations carries risks, such as potential leakage due to crustal movements, which could undermine the effectiveness of CCS. Additionally, capture and storage technologies are expensive. Moreover, direct air capture (DAC) is a technology that removes CO_2_ from the atmosphere. The technology removes CO_2_ directly from ambient air using high-powered fans to draw air into a processing facility, where CO_2_ is separated through a series of chemical reactions before being compressed and stored [12]. Meanwhile, capturing CO_2_ from the air is the most expensive form of carbon capture due to its low concentration in the atmosphere compared to flue gases from power plants or industrial facilities.

As natural and cost-effective carbon-capturing and permanent storing methods, afforestation and reforestation offer a sustainable alternative to industrial carbon capture methods. Afforestation converts abandoned and degraded agricultural land into forests, while reforestation restores tree cover in deforested areas [13,14]. Statistics show that natural forest regrowth could absorb up to 8.9 billion metric tons of CO_2_ annually by 2050, offsetting approximately 23% of global emissions [15].

### 1.2. Research Gap and Objectives

Recent research has increasingly explored sustainable construction materials to mitigate climate change impacts. Meng et al. [16] provided an in-depth analysis of recent advancements in carbon sequestration within concrete materials, focusing on the role of carbonation curing technologies in conventional Portland cement (PC)-based materials. The CO_2_ absorption capacity of different binder compositions has been investigated [17], including Portland cement, pozzolanic materials, and alternative non-cementitious binders, to assess their potential for carbon sequestration. Also, Sangmesh et al. [18] explored agricultural waste as a sustainable alternative to reduce reliance on cement, indirectly contributing to CO_2_ reduction. Additionally, Bjånesøy et al. [11] offered valuable insights into biogenic carbon sequestration and storage (CSS) within urban built environments, though their focus was predominantly on biogenic materials, leaving significant gaps regarding the role and efficiency of non-biogenic materials in capturing, storing, and reducing CO_2_.

Furthermore, studies by Song et al. [6] and Ahmed et al. [19] have identified biochar, derived from biomass waste, as a sustainable additive for reducing carbon emissions in cement-based materials; however, broader applicability and integration strategies remain underexplored. Although Arehart et al. [8] provided a comprehensive review of carbon sequestration in buildings, particularly the concept of “buildings as carbon sinks” and quantification methodologies at varying scales, they did not sufficiently address detailed assessments of specific construction materials and their practical applications in carbon management. Consequently, despite the existing body of literature, substantial gaps persist, particularly concerning integrated studies that concurrently evaluate multiple construction materials and techniques capable of capturing, storing, and reducing CO_2_. Addressing these gaps through targeted research will be critical for advancing the development and implementation of multifunctional, low-carbon, climate-resilient built environments.

The study aims to provide a comprehensive analysis of methodologies and strategies applied in the built environment (BE) for capturing, storing, and reducing CO_2_ emissions. By focusing on methodological approaches rather than solely on specific materials, the review facilitates a holistic understanding of carbon management practices within the BE. The specific objectives of this review include:

Providing a state-of-the-art overview of the principles and mechanisms underlying carbon capture, storage, and reduction methodologies applicable to the built environment.

Critically evaluating existing methodologies and strategies employed in construction processes and built infrastructure to capture, store, and reduce CO_2_ emissions.

Systematically classifying practical implementations of carbon management strategies within various built environment applications.

Assessing the potential and feasibility of integrating novel methodologies, such as direct air capture, into construction processes and existing infrastructure.

Identifying critical knowledge gaps and proposing strategic research directions to guide future advancements and enhance the effectiveness of carbon management strategies in achieving sustainable, low-carbon built environments.

The study is structured as follows: Section 2 offers an overview of carbon classification principles in the BE, addressing carbon capture in Section 2.1, where various mechanisms such as photosynthesis, concrete passive carbonation, and microbial biomineralization are examined. Section 2.2 explores techniques, including carbonation curing, accelerated mineral carbonation, and using construction and demolition waste. Section 2.3 focuses on minimizing CO_2_ emissions using alternative materials. Section 3 dips into various materials with the potential for direct CO_2_ capture, although many are not yet widely adopted in construction. Section 4 identifies key research gaps and outlines future directions to promote a carbon-neutral built environment. Finally, Section 5 summarizes the key findings and provides concluding remarks on the role of sustainable materials in achieving a low-carbon built environment.

## 2. Carbon Capture, Storage, and Reduction Principles in the Built Environment

**Figure 2:**
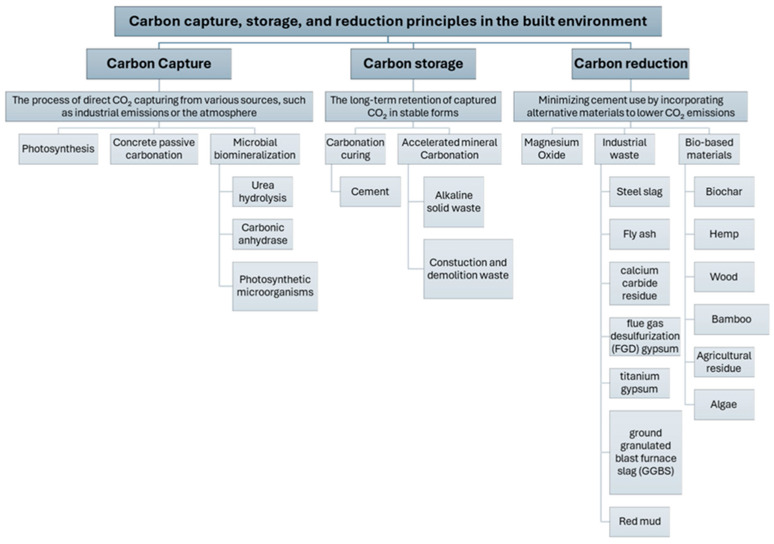
Overview of carbon capture, storage, and reduction pathways in the built environment. The figure illustrates key processes and materials involved in each strategy.

This section outlines the principles, mechanisms, and materials associated with carbon capture, storage, and reduction in the built environment. Figure 2 provides a visual summary of the processes under each strategy, and the following subsections provide a detailed discussion of each one.

### 2.1. Carbon Capture

Carbon capture refers to trapping carbon dioxide (CO_2_) from various sources, such as industrial emissions or the atmosphere, to mitigate its impact on climate change. This process is crucial in reducing greenhouse gas concentrations and can be integrated into construction materials and environmental applications. Carbon capture can occur naturally through biological processes or be engineered using chemical and mineralization techniques. The following subsections delve into key mechanisms: photosynthesis in Section 2.1.1, where plants and bio-based materials absorb CO_2_; concrete passive carbonation, in Section 2.1.2, which involves CO_2_ reacting with cementitious materials to form stable carbonates; and microbial biomineralization in Section 2.1.3, where a microbial-induced process that converts CO_2_ into mineral deposits, enhancing durability and sustainability in construction materials.

#### 2.1.1. Photosynthesis

Photosynthesis is a natural carbon-capturing mechanism related to bio-based construction materials. It allows plants to convert carbon dioxide (CO_2_) from the atmosphere into biomass using sunlight as an energy source. This biological process supports plant growth and effectively sequesters carbon, locking it into its structure for extended periods. Materials derived from biomass, such as wood products, agricultural residues, and biomass waste, exhibit remarkable potential for incorporating photosynthetic carbon sequestration into sustainable construction practices. These bio-based materials serve as carbon sinks, storing CO_2_ captured during their growth phase. Biochar is a prime example of a material leveraging photosynthesis for carbon capture. As a carbon-rich byproduct of biomass pyrolysis under controlled oxygen conditions, biochar stabilizes atmospheric CO_2_ absorbed during photosynthesis, storing it long-term [19]. According to the IPCC, producing one ton of biochar offsets between −2.0 and −2.6 tons of CO_2_, underscoring its significant potential to combat climate change [20]. Hemp-based building materials further illustrate the power of photosynthesis in carbon sequestration. During growth, hemp sequesters substantial amounts of CO_2_, resulting in a negative carbon footprint when used in construction. A life cycle assessment (LCA) by Rivas-Aybar et al. [21] demonstrated that hemp-based boards achieve a carbon footprint of −2.302 kg CO_2_ eq/m^2^, significantly outperforming traditional gypsum plasterboards. Marine macroalgae, such as seaweed, also play a vital role in photosynthetic carbon sequestration, containing an average of 25–30% carbon by dry weight [22]. Their rapid growth in nutrient-rich environments makes seaweed biomass a renewable carbon-fixing resource. Scardifield et al. [23] demonstrated this potential by using residual biomass from Ulva ohnoi, a green seaweed, to develop sustainable bio-masonry products, showcasing the dual role of seaweed in sequestering CO_2_ during growth and its application as a sustainable construction material. Wheat straw is a biogenic material that has received significant attention. A cubic meter of straw stores 129.25 kgCO_2_ eq [24]. Furthermore, when used as an insulation material, it has been reported to have close to −60 kgCO_2_e per 1 square of material at a sufficient thickness to achieve a U value of 0.14 W/m^2^K. Detailed materials derived from bio-based materials captured CO_2_ by the photosynthesis mechanism and used in the built environment are illustrated in Table 1.

#### 2.1.2. Concrete Passive Carbonation

While concrete structures made of cementitious materials are a significant contributor to anthropogenic CO_2_ emissions, they are a potential carbon sink. Despite its slow nature, passive carbonation or weathering carbonation describes the process of carbon uptake in cementitious materials in which atmospheric carbon dioxide reacts with hydration products to form calcium carbonate. Alkaline compounds in cement, such as calcium hydroxide (Ca(OH)_2_) and calcium silicate hydrate (C-S-H), react with atmospheric CO_2_ over the material’s lifecycle [41]. Various national studies, for example, in Ireland and Sweden, have estimated carbon uptake by concrete structures to range from 75 to 125 kg of CO_2_ per ton of cement over a 100-year service life, potentially offsetting up to 16% of cement production emissions [42,43]. In China [44], carbon uptake in 2021 was estimated at 426.77 Mt CO_2_, contributing to national and global land sink estimates. While concrete structures can act as long-term carbon sinks through passive carbonation, global estimates of this effect vary widely. A key uncertainty lies in calculating the total exposed concrete surface area, which significantly influences uptake projections. Therefore, further research is needed to accurately assess the role of passive carbonation at scale [42,43,44,45,46].

Atmospheric CO_2_ reacts with calcium hydroxide (a by-product of the hydration process) to form calcium carbonate (calcite) (Equation (1)) [47], densifying the material and reducing porosity. However, excessive CO_2_ exposure can decompose C-S-H (Equation (2)), a critical component of cement’s binding matrix, leading to carbonation shrinkage and increased porosity.

Ca(OH)_2_ + CO_2_ → CaCO_3_ + H_2_O (1)

C-S-H + CO_2_ → CaCO_3_ + SiO_2_ + H_2_O (2)

#### 2.1.3. Microbial Biomineralization

Microorganisms capable of performing mineral carbonation have emerged as powerful tools for sustainable applications, particularly in geoengineering and construction materials. In the presence of calcium or magnesium ions, carbon-capturing bacteria can adsorb and transform CO_2_ into carbonates, facilitating the environmentally friendly process of microbial carbonization. This process, often mediated by metabolic pathways like ureolysis [48], carbonic anhydrase activity [49,50,51], and photosynthetic bacteria such as cyanobacteria [52], has been widely studied for its potential to induce calcium carbonate precipitation (MICP). MICP offers innovative solutions for CO_2_ sequestration and creates valuable materials for soil improvement [49], durability enhancement in cementitious structures [50], and novel uses within the BE.

##### Urea Hydrolysis

The urea hydrolysis pathway is one of the most studied microbial metabolic processes for calcium carbonate (CaCO_3_) precipitation [51]. This pathway is primarily driven by urease, an enzyme produced by ureolytic bacteria such as *Sporosarcina pasteurii*. Urease catalyzes the breakdown of urea (CO(NH_2_)_2_) into ammonia (NH_3_) and carbamate (NH_2_COO^−^) in an aqueous environment. The production of hydroxide ions (OH^−^) raises the pH, creating conditions favorable for CaCO_3_ precipitation, while the negatively charged cell surface of bacteria helps attract calcium ions, further promoting mineralization. A series of natural reactions then occurs. Finally, the carbonate ions (CO_3_^2−^) react with calcium ions (Ca^2+^) in the environment, leading to the precipitation of calcium carbonate (CaCO_3_), which can take forms such as calcite or vaterite. However, the high cost of urea and calcium sources presents a significant challenge to the practical application of urease-based calcite precipitation in field settings. Additionally, the release of ammonia (NH_3_) during the process raises environmental concerns due to its potential impact on pollution.

##### Carbonic Anhydrase

In contrast, bacteria that produce carbonic anhydrase can efficiently capture CO_2_, helping to reduce atmospheric CO_2_ levels. The carbonic anhydrase (CA) activity pathway is an enzymatic process that plays a critical role in CO_2_ sequestration and calcium carbonate (CaCO_3_) precipitation. Carbonic anhydrase is a highly efficient metalloenzyme found in various microorganisms, including bacteria like *Bacillus subtilis*, and it catalyzes the reversible hydration of carbon dioxide (CO_2_) into bicarbonate ions (HCO_3_^−^). This reaction is crucial in environments with calcium or magnesium ions, as it enables the formation of carbonate minerals under alkaline conditions.

##### Photosynthetic Microorganisms

Photosynthetic microorganisms, such as cyanobacteria and microalgae, can promote carbonate precipitation through their unique metabolic processes [52]. During photosynthesis, CO_2_ is consumed to produce organic compounds and oxygen, which re-establishes the carbonate equilibrium. This promotes the conversion of bicarbonate into CO_2_ and hydroxide ions, increasing alkalinity and enabling calcium carbonate precipitation. The coupling of photosynthesis with carbonate precipitation significantly enhances the CO_2_ fixation efficiency of these microorganisms, making them a promising solution for carbon sequestration and mineralization. Microbial-induced calcium carbonate precipitation (MICP) has been extensively studied and applied across various fields, as highlighted by notable studies [53]. Gilmour et al. [51] developed an innovative approach to carbon capture by genetically engineering *Bacillus subtilis* to express previously uncharacterized carbonic anhydrase (CA) enzymes from *Bacillus megaterium*. This engineered bacterium successfully sequestered CO_2_ and converted it into calcium carbonate (CaCO_3_), a key material for sustainable construction. The process demonstrated a significant reduction in CO_2_ levels (from 3800 ppm to 820 ppm) and produced calcite and vaterite minerals, confirmed via X-ray diffraction and scanning electron microscopy. By incorporating the bacterium SAML2018, capable of both ureolysis and CO_2_ hydration via urease and carbonic anhydrase enzymes, the study demonstrated significant improvements in CO_2_ capture and material performance [50]. The compressive strength value of carbonation-cured specimens was higher than that of water-cured specimens. This is attributed to promoting the hydration of stable C2S under carbonation conditions and a large amount of CaCO_3_ in a stable state. The obtained results implied the ability of MICP to repair concrete cracks. Yu et al. [49] focused on microbial carbonization for dust control, using *Streptomyces microflavus* to transform CO_2_ into stable calcite that binds sand particles, outperforming *Paenibacillus mucilaginosus* in terms of efficiency and mechanical strength. Together, these studies showcase the diverse potential of microbial processes in CO_2_ capture, environmental remediation, and the development of sustainable construction materials. Table 2 provides a summary of CO_2_ capture by microbial biomineralization.

### 2.2. Carbon Storage

Carbon storage refers to the long-term retention of captured CO_2_ in various forms to prevent its release into the atmosphere. In the built environment, carbon storage is integrated into construction materials through carbonation curing, where CO_2_ is absorbed into the concrete, and accelerated mineral carbonation converts CO_2_ into stable carbonates. The following subsections explore key storage mechanisms, including Section 2.2.1, where CO_2_ is absorbed during the early setting of concrete to improve strength and store carbon; accelerated mineral carbonation, in Section 2.2.2, where CO_2_ reacts with alkaline industrial waste to form stable carbonates; and the use of construction and demolition waste (CDW), in Section 2.2.3, which offers a dual benefit of waste valorization and permanent CO_2_ sequestration through mineral reactions.

#### 2.2.1. Carbonation Curing

Various carbonation procedures have been developed to enhance carbonation efficiency, particularly given the low atmospheric CO_2_ concentration (~0.041%) [54]. Early-age carbonation treatment has demonstrated a positive impact on the strength development of concrete by densifying its microstructure [55,56]. Carbonation curing, typically conducted shortly after casting, involves exposing early-age concrete to high concentrations of CO_2_. During this process, calcium hydroxide reacts with dissolved CO_2_ in the pore solution, precipitating calcium carbonate. Carbonation curing utilizes high-concentration CO_2_ sources, including pure CO_2_ and flue gas. While pure CO_2_ offers a significantly higher concentration (e.g., 99% compared to 20% in flue gas), flue gas is often more economical. This process, generally completed within the first 24 h of concrete mixing, enables CO_2_ to react with cement phases and hydration products, forming stable minerals such as calcium carbonate (CaCO_3_) and silica (SiO_2_), thus contributing to carbon sequestration. Although both weathering carbonation and carbonation curing facilitate carbon sequestration, they are fundamentally different. Weathering carbonation is a passive, long-term process, whereas carbonation curing is an active, short-term approach, typically completed within a day. Furthermore, the reactions in weathering carbonation predominantly involve calcium hydroxide (portlandite), which reacts with atmospheric CO_2_ to produce calcium carbonate, as described in Equation (1).

#### 2.2.2. Accelerated Mineral Carbonation

**Figure 3:**
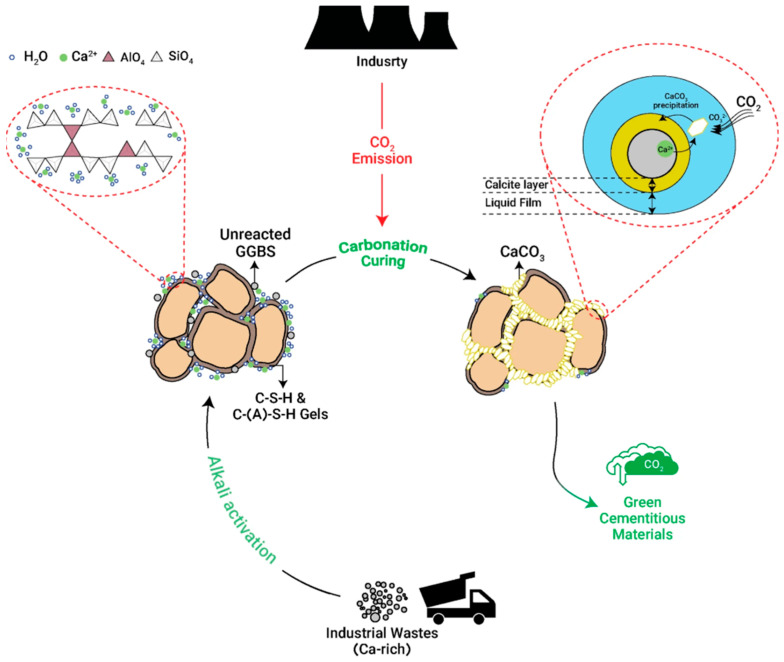
Schematic diagram of the accelerated carbonation process applied to alkali-activated industrial wastes. The process involves the dissolution of CO_2_ into pore water, forming carbonate ions that react with calcium and magnesium oxides present in the waste materials to precipitate stable carbonates such as calcite [65].

Mineral carbonation is a promising method for CO_2_ storage that involves the fixation of CO_2_ through reactions with alkaline and alkaline-earth oxides, such as magnesium oxide (MgO) and calcium oxide (CaO) [57], as shown in Figure 3. These chemical reactions create stable carbonates, including magnesium carbonate (MgCO_3_) and calcium carbonate (CaCO_3_). This process mimics and accelerates the natural geological process of rock weathering, providing a means for long-term carbon storage [54]. In addition to naturally occurring oxides and magnesium, iron, and calcium silicates, alkaline solid wastes from industrial processes such as municipal waste incineration ash, coal combustion by-products, cement production residues, and steel slag are suitable feedstocks for mineral carbonation. These materials offer a faster reaction rate and higher carbonate conversion efficiency than natural minerals and require minimal energy input, making them highly practical for CO_2_ sequestration [58].

Using industrial by-products and construction and demolition waste materials in mineral carbonation provides dual benefits: effective waste management and reducing greenhouse gas emissions. Numerous studies have demonstrated the feasibility of carbonating a wide range of industrial wastes, including fly ash [59,60,61,62,63], Ground Granulated Blast Furnace Slag [64,65,66], steel slag [57,67,68,69,70], calcium carbide residue [71], red mud [72], FGD-gypsum [73], titanium gypsum waste [74], and construction and demolition waste [75,76,77,78,79,80,81], further highlighting the potential of this approach for creating sustainable construction materials while addressing CO_2_ emissions. Furthermore, Magnesium oxide (MgO) cement binders are gaining attention as sustainable alternatives to traditional Portland cement due to their ability to store CO_2_ through mineral carbonation [82,83,84,85,86,87,88].

Equations (3) and (4) summarize the general carbonation reaction of Ca and Mg-rich silicates and oxides, respectively.

XSiO_3_ + CO_2_ → XCO_3_ + SiO_2_ (3)

(4) $$XO+CO_{2}\to {XCO}_{3}$$

where X corresponds to either Ca or Mg.

This Mineral carbonation process can be categorized into direct carbonation, aqueous carbonation, and carbonation during mixing, each offering distinct advantages based on reaction conditions and material composition.

##### Direct Carbonation

Direct carbonation involves alkaline-rich materials, such as steel slag and fly ash, to concentrated CO_2_ at elevated pressures and concentration under controlled environments, expediting the conversion of oxides into stable carbonates. Various studies have explored direct carbonation for CO_2_ sequestration and material enhancement, differing in curing conditions, CO_2_ exposure settings, and pressure levels. Razeghi et al. [65] applied carbonation to alkali-activated slag-stabilized soil after 7 days of initial curing, exposing it to 100, 200, and 300 kPa CO_2_ for 1 h in a sealed chamber, finding that excessive pressure reduced strength. In contrast, Kravchenko et al. [68] carbonated steel slag samples without prior extended curing, placing them on a plastic grid in a sealed chamber at 0.15 MPa CO_2_ for 24 h, with polyvinyl alcohol (PVA) improving CO_2_ retention. Feng et al. [89] investigated carbonation resistance in geopolymers, sealing five out of six surfaces to ensure that CO_2_ could only diffuse into the interior of the specimen from the exposed face and exposing the samples to 20% CO_2_ at 70% humidity and 20 °C, with exposure durations ranging from 12 h to 28 days. Lastly, Xian et al. [57] studied early-age carbonation curing in cement-free concrete made from steel slag, applying carbonation immediately after casting in either a near-ambient pressure (1.4 kPa) inflatable enclosure or a high-pressure (500 kPa) sealed chamber, both for 24 h before transferring the samples to a moisture room for further hydration curing.

##### Aqueous Carbonation

Aqueous carbonation process where a constant flow of CO_2_ is bubbled into an aqueous solution containing alkaline, leading to carbonate precipitates. Compared to direct carbonation, aqueous carbonation enhances CO_2_ uptake by improving ion mobility in liquid media, eliminating diffusion limitations, and significantly accelerating the reaction. Martos et al. [83] developed a magnesium carbonate trihydrate (MgCO_3_·3H_2_O) material by bubbling CO_2_ (10%) into a NaOH solution, which then reacted with Mg-rich brines, achieving an 85–90% Mg precipitation yield at room temperature in ~3 h. In contrast, Lin et al. [74] applied an indirect aqueous carbonation method to titanium gypsum, first extracting Ca^2+^ using ammonium acetate before bubbling CO_2_ at 100 mL/min, resulting in 97.28% vaterite CaCO_3_ conversion within 20 min. Han et al. [64] dissolved CO_2_ in a 3M NaOH solution, producing a CO_2_-activated alkali solution that instantly carbonated alkali-activated slag (AAS). Other alkaline materials, such as FGD gypsum [73] and CFBC ash [90], have high Ca^2+^ availability, making them practical for CO_2_ sequestration through aqueous carbonation.

##### Carbonation During Mixing

Luo et al. [62] explored CO_2_ injection during the mixing stage of fly ash (FA) blended cement pastes to enhance early hydration and mechanical properties. High-purity CO_2_ (99%) was injected into fresh cement pastes during the final 60 s of mixing at varying doses (0.3%, 0.6%, 0.9%, and 1.2% by cement weight). The study found that lower CO_2_ doses (0.3–0.6%) promoted early-age strength (3.3–8.2% increase at 1–3 days) by forming CaCO_3_, which acted as a nucleation site for hydration.

#### 2.2.3. Construction and Demolition Waste

As discussed in Section 2.2.2, construction and demolition waste (CDW) is highly suitable for mineral carbonation due to its abundant alkaline materials, such as calcium, which react with CO_2_ to form stable carbonates, enabling waste valorization and carbon sequestration. In 2020, global solid waste generation reached 2.24 billion tons, with CDW comprising at least 30% of this total [91]. Recent research highlights its potential as a sustainable resource in construction, particularly for cement replacement and CO_2_ storage [76,77].

Kravchenko et al. [76] explored waste concrete powder (WCP) as a carbon storage material and cement substitute, evaluating different scenarios through life cycle assessment (LCA). The findings showed that 8 h carbonation at 0.15 MPa CO_2_ achieved an optimal uptake of 42.7 kg CO_2_-e per tonne, resulting in a carbon-negative global warming potential (GWP) of −4.22 kg CO_2_-e. While carbonated WCP had limited CO_2_ uptake as a cement replacement, uncarbonated WCP reduced the carbon footprint by 16% when replacing 20% of cement in concrete mixes. Similarly, the accelerated carbonation process developed a zero-cement hollow block with 75% recycled concrete fines [77]. The carbonation of the hollow blocks resulted in a CO_2_ uptake of approximately 100 kg CO_2_/ton of product, with an average compressive strength of 15.4 MPa at the lab scale and 6.4 ± 0.2 MPa at pilot production, meeting the minimum compressive strength of 5 MPa needed for hollow blocks that can be used as non-bearing separation walls.

**Figure 4:**
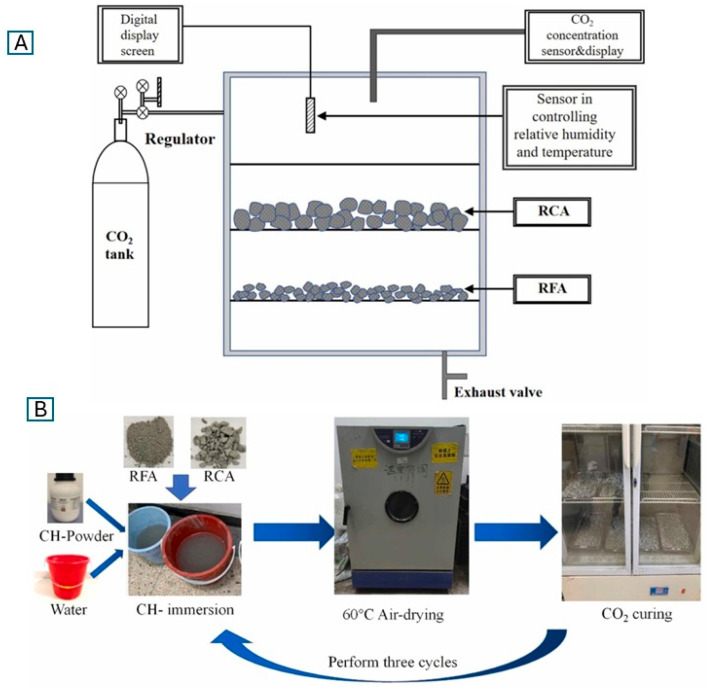
Illustration of the CO_2_ curing procedures applied to recycled aggregates in this study [78]. (**A**) Schematic diagram of the standard CO_2_ curing chamber setup, where recycled coarse aggregates (RCA) and recycled fine aggregates (RFA) are exposed to controlled CO_2_ concentration, temperature, and relative humidity using an airtight system equipped with sensors and a digital display. (**B**) Process flow of the Ca(OH)_2_ immersion-assisted CO_2_ curing method (HH group): recycled aggregates are first soaked in a saturated calcium hydroxide solution, followed by air-drying at 60 °C and then CO_2_ curing. This cycle is repeated three times to enhance the carbonation potential.

CDW can also be used to produce carbonated recycled concrete aggregates (RCA) [75,77,78,79,80], offering permanent CO_2_ storage while reducing reliance on natural aggregates, which are increasingly scarce. The research [77] demonstrated that carbonating RCA with size range from 4 to 16 mm at atmospheric pressure, with CO_2_ concentrations as low as 10% and temperatures of 60 °C, significantly reduced water absorption and increased aggregate hardness, enabling up to 50% replacement of natural aggregates without compromising concrete strength. Recycled brick powder (RBP) was also investigated as a partial sand replacement, where carbonation improved lime reactivity, accelerated hydration by 4–5 h, and enhanced compressive strength by 19–21% compared to non-carbonated RBP [75]. The CO_2_ uptake of pre-carbonated RBP (16–17%) was significantly higher than the 10.7% observed in control samples. To improve recycled aggregate concrete (RAC) durability, particularly under freeze–thaw conditions, Liang et al. [78] compared three CO_2_ curing methods: standard curing, pre-saturated calcium hydroxide Ca(OH)_2_ immersion, and whole-specimen CO_2_ curing in Figure 4. All methods enhanced peak stress and elastic modulus (12.3–13.7% and 19.2–31.5%, respectively), with Ca(OH)_2_ immersion achieving the best performance. Fernández et al. [80] further demonstrated that carbonated water as kneading water in cement-based materials (CBMs) improved early-age strength retention and long-term durability, particularly when combined with recycled masonry aggregates (RMA).

Beyond structural applications, CDW can enhance urban ecosystems. Fan et al. [92] evaluated waste building material substrates (WBMS) in green roofs over a year-long study, showing superior carbon sequestration (12.8 kg C/m^2^/year), 1.1 times higher than local natural soil (LNS). WBMS recycled CDW and improved urban ecosystem services, highlighting its dual role in waste management and climate change mitigation.

**Figure 5:**
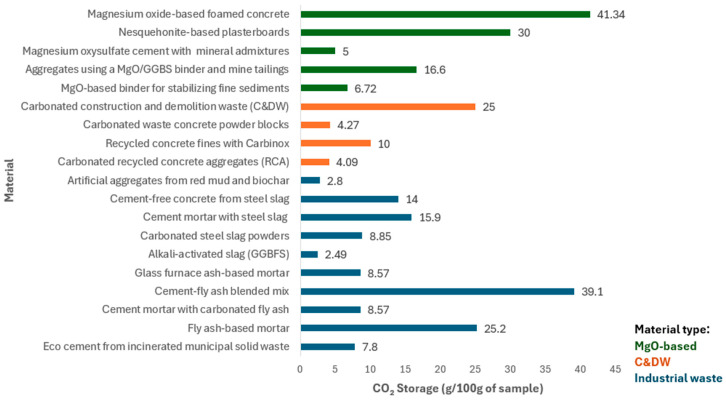
CO_2_ storage capacity (g per 100 g of sample) of various materials used in the BE. Materials are grouped by type: MgO-based materials (green), construction and demolition waste (C&DW) (orange), and industrial waste-derived materials (blue). References for each material corresponding to the order of materials shown in the figure, from top to bottom, within each category: (MgO-based: refs. [82,83,84,85,86]), (C&DW: refs. [60,75,76,77]), (Industrial waste: refs. [57,59,60,61,63,64,67,69,72,93]).

To provide a comparative perspective on CO_2_ storage performance, Figure 5 shows the CO_2_ uptake capacities of various construction materials discussed throughout Section 2.2. The materials are grouped into three categories based on their origin: MgO-based materials, construction and demolition waste (C&DW), and industrial waste-derived materials. The reported values reflect results obtained under varying carbonation methods and conditions, including direct carbonation, aqueous carbonation, and carbonation during mixing. These differences in process, pressure, duration, and CO_2_ concentration contribute to the observed variation in uptake performance across materials.

### 2.3. Carbon Reduction

Carbon reduction in construction primarily focuses on minimizing the use of traditional cement products, which are major contributors to CO_2_ emissions. Alternative materials include Magnesium oxide binders Section 2.3.1, industrial waste materials Section 2.3.2, and bio-based materials Section 2.3.3, which promote recycling and sustainable resource use. The following subsections explore these approaches in detail, highlighting their potential to drive sustainable construction practices.

#### 2.3.1. Magnesium Oxide

Another alternative approach towards sustainable construction materials is through MgO incorporation. The production of MgO requires lower temperatures than the conversion of CaCO_3_ into ordinary Portland cement (OPC), resulting in significant energy savings and positioning MgO-based cement as a promising option for sustainable cement production [94]. Additionally, MgO’s capacity to absorb CO_2_ from the atmosphere and form various carbonates and hydroxy carbonates aligns with the concept of “carbon-neutral” cement, capable of offsetting nearly the same amount of CO_2_ emitted during its manufacturing process. Magnesia (MgO) can be produced not only through the calcination of magnesia-based minerals (e.g., magnesite, dolomite, or serpentine) but also via the alkaline precipitation of brucite (Mg(OH)_2_) from seawater or magnesium-rich brine [83]. Additionally, MgO obtained from seawater/brine has been proven to outperform MgO obtained from the magnesite calcination with higher purities and reactivities because of the larger specific area (SSA) [95].

Li et al. [84] investigated oxysulfate (MOS) cement, which integrates fly ash (FA) and ground granulated blast furnace slag (GGBFS) to enhance sustainability. When subjected to accelerated carbonation for 24 h, the material achieved 5% CO_2_ uptake, improving early-age compressive strength due to the formation of hydrated magnesium carbonates (HMCs). The study showed that the CO_2_ curing treatment improved compressive and flexural strength. However, excessive FA and GGBFS additions were found to reduce compressive strength, likely due to the diminished formation of the 5·1·7 phase (Mg(OH)_2_·MgSO_4_·7H_2_O). This highlights the need for an optimal balance in the composition of MgO-based cementitious materials. Similarly, Gonçalves et al. [87] explored MgO as a partial OPC replacement in cementitious mortars, demonstrating that MgO promotes the formation of magnesium hydroxide Mg(OH)_2_, which reduces shrinkage strain by up to 80% due to its expansive properties.

Martos et al. [83] developed a carbon capture and utilization (CCU) process that effectively captures CO_2_ from power plant flue gas (~10% CO_2_ concentration) and converts it into nesquehonite (MgCO_3_·3H_2_O)-based materials. This sustainable alternative to gypsum plasterboard has a CO_2_ capture efficiency of over 99%, demonstrating its potential for large-scale application. This material, containing over 30% CO_2_ by weight, can be hardened into plasterboard-like products using thermal activation or forced conversion, further enhancing its eco-efficiency. Additionally, precipitated calcium carbonate (PCC) by-products from the process can be used as aggregates or fillers, improving material sustainability.

A novel MgO-based material, Magnesium Oxide Carbon Sequestration Foamed Concrete (MCFC), was introduced by [82] as an innovative approach to reducing carbon emissions and enhancing CO_2_ capture. Unlike conventional concrete, foamed concrete contains air foam, which improves thermal insulation, lightweight properties, and fluidity. In MCFC, reactive MgO replaces OPC, and CO_2_ foam replaces conventional air foam, resulting in a material with superior carbon sequestration capacity. A Life Cycle Assessment (LCA) revealed that MCFC reduces carbon emissions by 50% compared to traditional foamed concrete, with a Global Warming Potential (GWP) of 246.18 kg CO_2_ eq/m^3^, significantly lower than 505.6 kg CO_2_ eq/m^3^ for OPC-based foamed concrete. Field applications in a road widening project in Suzhou, China, demonstrated that replacing PC-based foamed concrete with MCFC reduced CO_2_ emissions from 1000 to 470 tons, reinforcing its feasibility for large-scale infrastructure applications, particularly in non-structural components such as road embankments and lightweight concrete elements.

Beyond structural materials, MgO-based binders have been developed for environmental remediation and soil stabilization. Hwang et al. [85] designed a binder of 50% MgO, 5% Lime, 18% FA, and 27% BFS, which achieved a compressive strength of 11.9 MPa, comparable to OPC (12.2 MPa). This binder was used to stabilize dredged sediments, where it not only enhanced compressive strength to 4.78 MPa after 365 days but also sequestered 67.2 kg CO_2_ per ton of sediment over a year. These results highlight MgO’s effectiveness in carbon storage and environmental stabilization applications. Kim et al. [86] investigated MgO-GGBS binders for stabilizing mine tailings and heavy metals, a crucial area given the high carbon footprint of traditional Portland cement (PC) in mining applications. A binder composition of 50% MgO and 50% GGBS was found to be the most effective, with granules cured under 20% CO_2_ achieving a compressive strength of 4.71 MPa after 28 days and a CO_2_ sequestration capacity of 0.159 kg CO_2_/kg binder, outperforming other mixtures.

#### 2.3.2. Industrial Waste

Industrial by-products play a critical role in both carbon storage and reduction, providing sustainable alternatives to conventional cementitious materials while significantly lowering CO_2_ emissions. As discussed in Section 2.2.2, many industrial wastes such as steel slag, calcium carbide residue (CCR), ground granulated blast furnace slag (GGBS), and fly ash are rich in calcium oxide (CaO) and magnesium oxide (MgO). These oxides facilitate mineral carbonation to form stable carbonates, ensuring permanent carbon sequestration within construction materials.

Beyond their role in carbon storage, industrial by-products contribute to carbon reduction by replacing traditional clinker-based cement [96]. They are widely utilized as Supplementary Cementitious Materials (SCMs), Alkali-Activated Materials (AAMs), geopolymers, and aggregates, reducing reliance on energy-intensive cement production and enhancing the environmental performance of concrete. The following subsections will examine the specific applications of industrial by-products in carbon reduction.

Li et al. [59] examined municipal solid waste incineration (MSWI) fly ash (50%) as SCM, demonstrating that CO_2_ curing at 0.5 MPa for 2 h increased compressive strength to 12.3 MPa, 3.43 times higher than untreated mortars. CO_2_ capturing has also improved, rising from 17.2% to 25.2% at 2.5 MPa. Similarly, carbonation-treated circulating fluidized bed combustion (CFBC) ash enabled its use as a cement substitute, with 20% replacement maintaining strength and 25% replacement producing suitable foam concrete with optimal thermal conductivity and density [90]. Huseien et al. [97] explored high-volume fly ash (60%) combined with bottle glass waste nanoparticles (BGWNPs, 2–10%), resulting in reductions of 61.9% in CO_2_ emissions, 54.3% in energy consumption, and 50.6% in binder cost compared to OPC. High-calcium fly ash (HFA) has also been studied for carbonation curing, with Su et al. [61] achieving a CO_2_ uptake of 8.24%, alongside improved resistance to freeze–thaw cycles and sulfate attack. Similarly, Li et al. [67] found that reducing steel slag particle size from 112.6 μm to 22.4 μm doubled CO_2_ uptake (from 37.9 kg CO_2_/t to 88.5 kg CO_2_/t), as higher surface area facilitated ion leaching and carbonation efficiency.

Beyond SCMs, Alkali-Activated Materials (AAMs) or geopolymers provide low-carbon cement alternatives using aluminosilicate precursors activated with alkaline solutions. Alkali-activated slag binders using GGBFS have been shown to stabilize soil, with 20% binder content improving mechanical properties under accelerated curing [65]. Coffetti et al. [98] demonstrated that AAMs derived from GGBFS achieved compressive strength targets while reducing Global Warming Potential (GWP) by up to 75% in applications like structural plaster and pervious concrete. Similarly, reference [66] developed an eco-friendly cement binder using fly ash, blast furnace slag, rice husk ash, and aluminum foil, leading to a 55% CO_2_ emission reduction and 35% cost savings compared to OPC. In addition to fly ash and slag, other industrial residues such as waste glass and red mud are gaining traction as promising precursors for geopolymer-based construction materials. Red-mud-based geopolymers (RM-GM) convert industrial waste into low-carbon binders, achieving up to ~80 MPa compressive strength and 60–64% lower CO_2_ emissions than cement [99]. They also immobilize heavy metals (>80%) and are applicable in construction, soil stabilization, and environmental remediation. Additionally, waste glass is emerging as a viable alternative precursor for low-carbon geopolymer systems. Xiao et al. [100] propose a Portland cement-free, high-volume waste glass geopolymer composite, in which both glass powder (GP) and glass cullet aggregates are used to achieve up to ∼83 wt% waste glass in the total solid content.

Other industrial by-products, such as red mud from bauxite refining, offer additional applications, including artificial aggregates. Liu et al. [72] investigated artificial lightweight cold-bonded aggregates (ALCBAs) made from red mud and biochar, achieving CO_2_ uptake of 26.0–29.1 kg/ton under natural carbonation. The process involved CO_2_ reacting with alkaline minerals (calcium silicate, portlandite) to form stable carbonates, while biochar enhanced CO_2_ storage due to its porous structure. In infrastructure applications, red mud (RM) based cementitious materials were developed for semi-flexible pavement (SFP) as an eco-friendly alternative to traditional grouting materials [101]. Two types were evaluated: red mud-slag powder composite (RSCM) with 50% RM and 50% slag powder, and red mud-cement composite (RCCM) with 10% RM and 90% cement. RCCM exhibited superior properties that met the standards for asphalt pavement in heavy traffic. A Life Cycle Assessment (LCA) indicated significant reductions in energy consumption and Global Warming Potential (GWP) compared to conventional materials.

Several key factors governing the carbonation behavior of industrial wastes such as CO_2_ pressure, reaction duration, temperature, particle size, and w/b ratio. With respect to CO_2_ pressure, most studies show that moderate pressures enhance early carbonation by increasing CO_2_ dissolution, as seen in Li et al. [59] and Razeghi et al. [65]; however, excessively high pressures often reduce efficiency by causing rapid CaCO_3_ precipitation that blocks pores and limits further reaction, a trend also observed in Xian et al. [70] where ambient-pressure carbonation performed as well as high-pressure curing. Regarding temperature, moderate heating promotes ion dissolution and accelerates carbonation; however, temperatures above ~30–40 °C decrease CO_2_ solubility and weaken later-stage reactions [59]. Particle size plays a central role in governing the reactivity and carbonation efficiency of industrial wastes used as binder components. Finer particles offer a greater specific surface area, which enhances the dissolution of reactive Ca- and Mg-bearing phases and accelerates the formation of carbonates. However, excessively fine fractions can slow CO_2_ transport by reducing pore connectivity. For example, Li et al. [67] found that aqueous-carbonated steel slag with particle sizes ranging from 22.4 to 112.6 μm exhibits CO_2_ sequestration of 88.5–37.9 kg CO_2_/t, with finer particles showing ~25% higher Ca^2+^ release. Similarly, in converter-slag mortars cured under ambient CO_2_, optimal performance occurs at intermediate sizes (21.75–24.13 μm), which maximize strength (≈31 MPa) and CO_2_ uptake (15.9%) [69]. Moisture availability, reflected through water-to-binder ratio, curing humidity, or aqueous conditions, also follows a consistent trend as sufficient pore water is essential for CO_2_ hydration and ion transport, yet excessive water blocks gas pathways, while insufficient water limits dissolution [65,89].

A comprehensive summary of industrial wastes in carbon reduction, including their application, carbonation conditions, and mechanical performance, is presented in Table 3.

#### 2.3.3. Bio-Based Materials

Bio-based materials contribute to carbon reduction by capturing and storing atmospheric CO_2_ through photosynthesis while serving as low-carbon alternatives to conventional construction materials. This section explores the role of biochar, hemp, wood, bamboo, agricultural residues, and algae in carbon reduction and long-term CO_2_ storage, highlighting their potential in sustainable built environments.

##### Biochar

Biochar, which is produced through the pyrolysis of biomass such as crop residues, livestock manure, and sewage sludge, is a carbon-negative material that offers two main benefits. First, it sequesters carbon that is captured during photosynthesis. Second, it can be utilized as a construction material in the BE. Additionally, biochar is an effective carbon dioxide adsorbent due to its high porosity. Biochar has different applications, a, including the application in cementitious mortars and concrete [25,36], fillers [19,30], aggregates [20,34,72], and wastewater treatment absorbents [74].

**Table 3:** Comprehensive summary of industrial wastes in carbon reduction.

Material Mix	Waste Source	Compressive Strength (MPa)	Carbonation Condition	CO_2_ Uptake/Reduction	Application	Ref.
CCR-to-FA ratios: 50:50 and 30:70 20% steel slag (SL) tested as an alternative to CCR Water-to-binder ratios: 0.10, 0.15, 0.20	Calcium carbide residue (CCR): a byproduct from acetylene production using calcium carbide (CaC_2_) Coal-gasification fly ash (FA)	>18	CO_2_ applied at 0.2 MPa for 2 h and 6 h	~16.5–19.6 g CO_2_ absorbed per 100 g of binder	Solid and hollow bricks using alternative lime binders	[71]
Steel slag as a binder PVA powder as an activator Water-to-slag ratio: 0.18	Steel slag: a by-product of the basic oxygen steelmaking		CO_2_ applied at 0.15 MPa pressure for 24 h	Steel slag with 0.6, 0.8, and 1.0% PVA absorbs 2.9, 5.4, and 5.9% CO_2_ by weight of the slag, respectively	Steel slag-based binder	[68]
Poorly graded sand (SP) Ground Granulated Blast Furnace Slag (GGBS) as a binder (10%, 15%, 20%, and 25%) Constant water content: 13.5 wt% (relative to dry soil) Alkali activators: NaOH and Na_2_SiO_3_	GGBS: a byproduct from the ironmaking process in blast furnaces		CO_2_ applied at pressures of 100, 200, and 300 kPa for 1 h		Soil stabilization	[65]
90% commercial gypsum and 10% carbonated flue gas desulfurization (FGD) gypsum waste Water-to-solid ratio: 0.5	FGD gypsum: byproduct of flue gas desulfurization in power plants and industrial facilities	6.1 ± 0.6	Indirect carbonation by NaOH		Gypsum Panels	[73]
Red mud slag cementitious material (RSCM): 50% red mud, 50% limestone slag powder, Water-to-cement (W/C) ratio: 0.85 Red mud cement cementitious material (RCCM): 10% red mud, 90% cement, W/C ratio: 0.42 Alkali activator: NaOH	Red mud: industrial solid waste with high PH	RSCM: 9.5 MPa at 7 days RCCM: 18.88 MPa at 1 day		Red mud-based SFPs exhibit 90.87% lower global warming potential (GWP) compared to cement-based SFPs	Low carbon Semi-flexible pavement (SFP)	[101]
Fly ash (FA) and ground granulated blast furnace slag (GGBS) with FA/GGBS ratios: 0/100, 20/80, 40/60, and 60/40 Polyethylene (PE) fibers: 1%, 1.5%, 2%, and 2.5% Water-to-binder (W/B) ratios: 0.32, 0.34, 0.36, and 0.38 Alkali activators: Na_2_O and SiO_2_	GGBS: a byproduct of ironmaking in blast furnaces Fly ash: from coal gasification	At FA/GGBS ratio 40/60, 6% alkali content, W/B ratio 0.36, and 2% fiber: 82 MPa before carbonation 80 MPa after 7 days	CO_2_ concentration: 20 ± 0.2% Relative humidity: 70 ± 5% Temperature: 20 ± 2 °C Duration: 12 h, 36 h, and 3, 7, 15, and 28 days		Engineering geopolymer composites (EGC)	[89]
Red mud, cement (10%), and biochar (5%, 10%, 15%) Water-to-solid ratio: 0.2	Red mud: solid waste is produced when alumina is extracted from bauxite ore	1.37 to 1.95 at 28 days	CO_2_ generated via a reaction between citric acid and baking soda Duration: 36 h	Carbon uptake: 30.58–33.06 kg CO_2_ per ton	Artificial lightweight cold-bonded aggregates (ALCBAs)	[72]
AAS Structural plaster: GGBFS, calcareous filler, sand, 3 alkaline activators, water Pervious concrete: GGBFS, alkaline activators, natural sand, recycled aggregates, water, air entraining agent, and superplasticizer Alkaline activator: sodium metasilicate pentahydrate, potassium hydroxide, and sodium carbonate	GGBS: A byproduct of the ironmaking process in a blast furnace	Structural plaster: ≥10.8 (28 days) Pervious concrete: up to 20		AAS materials reduce the GWP from 50 to 75%	Alkali-activated slag (AAS) as a Sustainable binder for Pervious Concrete and Structural Plaster	[98]
GGBFS, NaOH alkali activator with different CO_2_ concentrations Activator-to-precursor ratio of 0.55, and the ISO standard sand-to-precursor ratio was 3.2	GGBS: A byproduct of the ironmaking process in a blast furnace	30 MPa at 28 days	NaOH activation under CO_2_ concentrations of 0, 0.5, 1.0, and 1.3 mol/kg	CO_2_ uptake up to 2.49%	Sustainable alkali-activated slag mortar	[64]
Eight mortar mixtures: Two mixtures with BOF or OHF slag, metakaolin, waterglass, and standard sand Replicated versions with slag aggregates replacing standard sand Vegetable oil added to all four variants Alkali activation using a potassium-based activator	Steel slag: a by-product of the basic oxygen steelmaking	94 at 28 days		Average GWP reduction: 52% Up to 74% reduction with vegetable oil addition.	Steel slags as binder compounds and aggregates in alkali-activated systems	[102]
OPC (50%) and fly ash (50) NaH_2_PO_4_ used as a stabilizer Water-to-binder (W/B) ratio: 0.18 to 0.46	Fly ash: municipal solid waste incineration	Up to 19 at 28 days	CO_2_ pressure: 0.5 to 2.5 MPa Temperature: 20–50 °C Duration: 2 h	CO_2_ uptake: Up to 25.2% by weight at 2.5 MPa CO_2_ pressure	SCM	[59]
Leaching solution derived from titanium gypsum Biochar absorbents made from leaching residue and peanut shells	Titanium gypsum: waste from the production of titanium dioxide by the sulfuric acid process		CO_2_ injected into the leaching solution at a flow rate of 100 mL/min	The carbonation efficiency reached 97.28%	Vaterite CaCO_3_, potential construction material Absorbent for wastewater treatment.	[74]
EAF steel slag and fine aggregates Water-to-binder (W/B) ratio: 0.15	Steel slag: Electric Arc Furnaces (EAF), a byproduct of the steelmaking process	30 MPa under high-pressure carbonation	Near-ambient pressure: 1.4 kPa CO_2_ for 24 h High pressure: 500 kPa CO_2_ for 24 h	AP-carbonation can reduce 224.7 kg CO_2_ per tonne of steel slag concrete	Steel slag concrete slabs and pipes	[57]
(OPC), fly ash (60%), and glass particles (BGWNPs) (2, 10%) Water-to-cement (W/C) ratio: 0.55	Fly ash: extracted from industry wastes as the main source of aluminum silicate BGWNPs: produced from recycled glass bottles using a top-down method	With 6% BGWNPs: 37.9 MPa at 28 days		61.9% reduction in CO_2_ emissions compared to conventional OPC	Sustainable concrete repair materials	[97]
Concrete with CFBC ash as admixture: Cement, carbonated CFBC, standard sand, and water Foam Concrete with CFBC ash as cement replacement: cement, gypsum, carbonated CFBC, Standard AE Agent, water	Fly ash: by-product generated from coal-fired power plants	Cement mortar: 5–12% improvement in initial strength Foam concrete: 2.1 MPa at 28 days	Optimal CO_2_ flow rate: 700 cc/min		Concrete SCM and cement replacement in foam concrete	[90]
Cement and steel slag (10%) Water-to-binder (W/B) ratio: 0.35	Steel slag powder sourced from a local steel-making plant	73.6 MPa at 28 days	Aqueous carbonation at 40 °C for 2 h	CO_2_ uptake: 37.9–88.5 kg per ton of steel slag	SCM in concrete	[67]
C&DW and a separate cement-fly ash mixture Water-to-cement (W/C) ratio: 0.4	C&DW: sourced from the demolition of a 100-year-old building Cement–fly ash mixture: collected from IIT-BHU premises		CO_2_ flow rate: 1 L/min Temperature: 35–40 °C Relative humidity: 60–80% Duration: 10, 15, 20, 25, and 30 h	Cement–fly ash mixture: 39.1% carbonation C&DW: 25% carbonation under optimal conditions	SCM	[60]
OPC, Carbonated High-Calcium Fly Ash (10, 30%), fine aggregates, and superplasticizer Water-to-binder (W/B) ratio: 0.4	High-calcium FA (HFA) a by-product of coal combustion		Fly ash treatment: CO_2_ concentration of 20% for 2 and 4 h Cement mortar curing: CO_2_ concentration of 20%, temperature of 20 ± 0.5 °C, relative humidity of 70 ± 2% for 12 h	4 h carbonation increased CO_2_ uptake to 8.57%	SCM in mortar suitable for Freeze–Thaw and sulfate attack	[61]
Fly ash, granulated blast furnace slag, silica source, rice husk ash, and aluminum foil (NaOH) as the alkali source	Class F fly ash: from Australian coal power plants GBFS: from iron refineries	Up to 20 MPa with 0.5% aluminum addition	Temperature: 25 °C Relative humidity: 65% CO_2_ concentration: 20% Duration: 3, 6, and 15 days	55% lower CO_2_ emissions compared to OPC	Green Non-Cementitious Binder	[66]
Cement, converter steel slag (80%) with various sizes, and sand Water-to-binder (W/B) ratio: 0.4	Converter steel slag: a by-product of the basic oxygen steelmaking	Up to 31.21 MPa	Ambient CO_2_ curing at: Temperature: 298 K CO_2_ concentration: 0.2 atm Pressure: 1 bar Relative humidity: 65% Duration: 3, 7, and 14 days	CO_2_ uptake up to 15.9%	SCM in mortars	[69]
Cement, fly ash (10%, 15%, and 20%) by weight of cement Water-to-binder (W/B) ratio: 0.5	Class-F fly ash (FA) was sourced from Deteng Mineral Processing Plant		CO_2_ injected during the fresh stage Injection occurred during the last 60 s of mechanical mixing Mixing speed: 188 rpm CO_2_ dosages: 0.3%, 0.6%, 0.9%, and 1.2%		SCM in cement paste	[62]
Cement (70%), IFA, GFA, and ground glass powder (15%) Water-to-binder (W/B) ratio: 0.4	IFA comes from municipal solid waste incineration plants. GFA comes from high-temperature gasification processes in waste-to-energy (WTE) plants.	Up to 29 MPa at 90 days	Aqueous carbonation process: Ashes mixed with water (L/S ratio: 10 mL/g) Slurry stirred for 1 h High-purity CO_2_ injected at 100 mL/min	GFA: 74.1% carbonation, 87.5 mg/g CO_2_ uptake IFA: 4.6% carbonation, 3.1 mg/g CO_2_ uptake	SCMs in cement mortar	[63]
Steel slag Water-to-binder (W/B) ratio: 0.15	Steel slag (SS): electrical arc furnace (EAF) slag generated in a Canadian steel manufacturing plant	24.8–33.9 MPa	Ambient pressure: 1.4 kPa, 99.9% CO_2_, duration: 2 to 24 h High pressure: 0.5 MPa, 99.9% CO_2_, duration: 2 to 24 h	AP carbonation-activated SS: Approx. 7.5 tons of CO_2_ absorbed per 100 tons of slag	Non-Cementitious Binder	[70]
Mix 1: 45% APC, 30% boiler ash, and 25% bottom ash Mix2: 36% APC, 29% boiler ash, 29% bottom ash, 5% Ca(OH)_2_, 1% SiO_2_ Water-to-binder (W/B) ratio: 0.1 Concrete blocks: 15% cement, Fine aggregate 48%, Coarse aggregate 32% Water-to-cement (W/C) ratio: 0.35	MSW incineration residues	33 MPa at 28 days	CO_2_ pressure: 152 kPa CO_2_ purity: 99.5% Duration: 2 h	Mix 2: 5% CO_2_ uptake	Non-Cementitious concrete blocks	[93]

As a soil amendment, biochar improves nutrient retention, water absorption, and microbial activity [103]. A global meta-analysis showed that biochar alone reduced global warming potential by 27.1%, while its combination with chemical fertilizers reduced net greenhouse gas emissions by 14.3% [104]. Study shows the impacts of biochar on soil evaporation and shrinkage. Biochar from wheat, corn, and rice straw increased soil water content by up to 132.3% and reduced cracking by up to 16.57% compared to untreated soil [105]. These improvements support long-term plant growth and enhance carbon dioxide uptake through sustained photosynthesis.

In the study by Zou et al. [20], a novel core–shell aggregate (CSA) was developed using biochar as the lightweight core, and a cementitious shell was formed through cold bonding. The optimized mix, MG80, replaced 80% of the cement with ground granulated blast furnace slag (GGBS), significantly lowering carbon emissions. In another study, artificial lightweight cold-bonded aggregates (ALCBAs) were developed using a high volume of red mud (90%) combined with varying dosages of biochar (0%, 5%, 10%, 15%) [72]. The aggregates demonstrated promising carbon sequestration potential, with CO_2_ uptake ranging from 26.0 to 29.1 kg per ton under accelerated carbonation conditions following 28 days of sealed curing.

In natural environments, the carbonation of cement is typically slow due to low atmospheric CO_2_ concentrations. However, biochar enhances carbonation efficiency in cementitious materials by promoting the production of hydration products and increasing carbon dioxide and water diffusion [38]. The carbonation process, primarily influenced by the cement matrix’s total surface area of pores, benefits from biochar’s abundant micropores and high specific surface area [37]. These properties improve biochar-cement composites’ water adsorption and retention. Additionally, biochar accelerates carbonation reactions by providing more reaction sites for CO_2_ and calcium hydroxide (CH) interaction, leading to increased CO_2_ absorption. Agarwal et al. [25] found that 5% biochar replacement in cementitious pastes achieved 11.3% CO_2_ uptake under accelerated carbonation curing (ACC) while increasing compressive strength by 37% and enhancing thermal insulation. Similarly, Ahmed et al. [19] reported that biochar derived from rice husk-coal blends absorbed 70% more CO_2_ than control samples, emphasizing its role in carbonation enhancement. In lightweight concrete, biochar contributes to carbon sequestration and environmental remediation. Biochar derived from peanut shells sequestered 541–980 kg CO_2_-e per ton while improving water resistance and heavy metal immobilization [36]. Additionally, nano-biochar from apricot kernel shells refined pore structures, increasing flexural fracture energy by 98% at 0.04% dosage, demonstrating its scalability for high-performance applications [30].

Beyond cement, biochar-plastic composites (PB composites) also exhibit high CO_2_ sequestration capacity. A study found that a 50:50 biochar-to-PET ratio sequestered 550 g CO_2_-e/kg of composite, while cement-based tiles emitted 152.96 g CO_2_/kg [28]. Similarly, biochar-magnesium oxysulfate cement (MOSC) particleboards achieved CO_2_ sequestration of 137 kg/ton, although mechanical strength remained challenging due to biochar’s high porosity [34].

Despite its advantages, higher biochar dosages (15–20%) can reduce material strength due to particle agglomeration and increased porosity [38]. In another study [37], biochar from corn straw (1–5%) improved compressive strength, refined pore structure, and increased internal CO_2_ uptake, reaching 5.05–6.19% CO_2_ absorption within 40 mm of the surface.

##### Hemp

Hemp-based materials are key in reducing carbon, offering high CO_2_ sequestration potential and sustainable construction applications. Industrial hemp absorbs 0.445 tons of CO_2_ per ton of dry stems during cultivation [106], which is permanently stored in lime-hemp concrete (LHC), hempcrete blocks, and hemp fiber composites, resulting in a negative carbon footprint.

Hemp fibers are valued for their hygrothermal and acoustic insulation properties, making them suitable for biodegradable insulation mats, fiber-reinforced concrete, cement paste, and mortars [107].

Hempcrete, a composite of hemp hurds, lime, and water, is recognized for its thermal insulation and coating applications. Rivas-Aybar et al. [21] found that hemp-based boards achieve a negative carbon footprint of −2.302 kg CO_2_ eq/m^2^, outperforming gypsum plasterboards, which release 10.59 kg CO_2_ eq/m^2^. Similarly, hemp-lime concrete walls exhibited emissions ranging from +10.165 kg CO_2_ eq/m^2^ in pessimistic scenarios to −9.696 kg CO_2_ eq/m^2^ under optimal conditions, with carbon sequestration in hemp shives and binder carbonation as critical factors [31]. Isopescu et al. [106] demonstrated that hempcrete masonry blocks can achieve a negative carbon footprint of −20.3168 kg CO_2_ eq, due to CO_2_ uptake during plant growth and carbonation over time, making them ideal for thermal insulation layers. Heidari et al. [40] further confirmed that untreated hempcrete sequesters −15.27 to −8.92 kg CO_2_-eq/m^2^, while sol–gel coated hempcrete exhibited a higher footprint (22.51 to 28.77 kg CO_2_-eq/m^2^). However, this remains significantly lower than traditional cavity walls, which have a footprint of 122.71 kg CO_2_-eq/m^2^.

Hempstone, a composite made from hemp stalks, artificial stone scraps, and resin, further demonstrates hemp’s versatility. Studies show that Hempstone reduces environmental impact by 60% compared to artificial stones, with a carbon sequestration rate of 1.67 kg CO_2_ eq per kg [108].

##### Wood

Wood and its byproducts play a significant role in carbon reduction, serving as low-carbon construction materials that store CO_2_ while replacing energy-intensive concrete and steel.

Gao et al. [109] developed mineralized nano-wood, integrating in situ CO_2_ capture with CaCO_3_ growth in delignified wood. The production methods include the fabrication of delignified wood by removing hemicellulose and lignin using an aqueous solution containing sodium hydroxide (2.5 M) and sodium sulfite (0.4 M). Then, the delignified wood (DW) was immersed in Ca(OH)_2_ solution for 4 h at room temperature and continuously passed through CO_2_ for 4 h to grow and accumulate micro-nano solid particles of CaCO_3_. The process achieved a high mineralization rate (39%), improved compressive strength (45.4 MPa), and thermal insulation (0.13 W/m·K), enhancing fire resistance and durability. This innovation highlights wood’s potential as a multifunctional, carbon-sequestering material.

Lignin, a complex aromatic biopolymer, provides structural integrity and rigidity to plants by binding cellulose and hemicellulose fibers [110]. As the second most abundant biopolymer on Earth after cellulose, lignin constitutes approximately 30% of wood by weight and contributes to its stiffness, rigidity, and natural antimicrobial properties [111].

Miao et al. [26] developed LignoBlock, a lignin-based biopolymer soil composite (BSC) for non-load-bearing applications such as partition walls and facades. This material achieved a net-negative carbon footprint (−0.14 to −0.23 kg CO_2_/kg), significantly outperforming lightweight concretes (0.17–0.34 kg CO_2_/kg) and OPC (0.21 kg CO_2_/kg).

Integrating wood waste into cementitious materials offers additional carbon reduction benefits. Kuoribo et al. [112] combined wood powder (WP) and glass powder (GP) in cement panels, achieving 18% cost savings, 3.3% CO_2_ reduction, and 70% annual energy savings compared to conventional panels. Ohenoja et al. [39] introduced a carbon capture and utilization (CCU) method using peat-wood fly ash, leveraging self-hardening properties to produce fly ash tiles while sequestering up to 150 kg CO_2_ per ton of ash.

Sustainably managed harvested wood products (HWPs) act as long-term carbon sinks while replacing high-emission materials. Chen et al. [113] demonstrated that structural panels and lumber significantly reduce GHG emissions, with 112 Mt CO_2_eq reductions over 100 years for panels and 93 Mt CO_2_eq for lumber. Non-structural panels required 39 years to achieve sequestration parity, while pulp and paper products contributed to emissions due to short lifespans and landfill decomposition.

##### Bamboo

Bamboo significantly contributes to carbon reduction thanks to its rapid growth, high carbon sequestration capacity, and increasing use as an alternative in sustainable construction materials. Species such as Moso bamboo can sequester up to 7.19 tons of CO_2_ per hectare annually [114], storing more carbon per unit than traditional timber.

With a long history as a construction material, modern advancements in industrial techniques have refined bamboo into engineered products like Glued Laminated Bamboo (GluBam), laminated bamboo lumber (LBL), and parallel strand bamboo (PSB) [115]. Among these, GluBam stands out for its mechanical properties and low carbon footprint, driving its adoption in structural applications and civil engineering projects. Liu et al. [114] demonstrated that engineered bamboo (GluBam) used in rural residential buildings reduces life cycle carbon emissions by 30.4% compared to reinforced concrete (RC) structures with superior thermal insulation properties. Similarly, Zhang et al. [33] conducted a comprehensive life cycle assessment (LCA) of a steel-glued Laminated Bamboo (GluBam) hybrid truss to evaluate its environmental and economic performance compared to an all-steel truss. The results revealed that the hybrid truss reduced carbon emissions to (4572.5 kgCO_2_eq) and energy consumption by (71.15 GJ) compared to steel. However, these benefits could be further optimized by including end-of-life energy recovery from bamboo combustion in life cycle assessments. A study [115] compared reinforced concrete (RC) and laminated bamboo lumber (LBL) in six-story residential buildings across five cities in China’s cold and severe cold regions demonstrated that substituting RC with bamboo reduces energy consumption by 3–5% and CO_2_ emissions by 7–20%.

##### Agricultural Residues

Using agricultural residues in the built environment presents an innovative solution for reducing carbon emissions while effectively utilizing waste.

Straw, the stem of cereal crops such as wheat and rice, has been used in construction for thousands of years. Straw has been used as a building material since the first settlements of ancient Egypt were constructed [116]. Various construction methods use straw as the structure and primary building enclosure material. Li et al. [35] demonstrated the potential of straw bale houses as a scalable low-carbon construction solution, particularly in rural areas. Among the structural forms analyzed, the wood frame straw bale house exhibited the lowest net embodied carbon emissions (ECE) of 19.09 kgCO_2_e/m^2^, achieving near carbon neutrality due to straw bales and wood’s high carbon storage potential. In contrast, masonry-concrete straw bale houses showed significantly higher ECE due to the reliance on high-carbon materials like cement and concrete. Di Luigi et al. [29] explored the development of sustainable insulation materials using wheat straw in advanced applications. By employing innovative additive manufacturing techniques, they transformed wheat straw into cellulose fibrils (CF) and combined them with silica aerogels to create high-performance insulation panels. These materials exhibited excellent thermal conductivity (0.036 W/m·K), enhanced structural integrity, and superior moisture resistance through in situ hydrophobic treatments.

Sugarcane bagasse ash (SCBA), a by-product of the sugar industry, has gained attention as a potential alternative to sand in crack repair mortar for rigid pavements [32]. The authors in their study showed that SCBA-based mortars improved compressive strength and reduced environmental impacts, with carbon footprint reductions of up to 24%. Including zeolite and recycled polypropylene fibers further enhanced carbon capture and reduced emissions by 50%.

Sisal fiber, one of the most utilized natural fibers, is easy to cultivate and highly accessible. With a global production of approximately 4.5 million tons annually, sisal fibers play a significant role in construction, where they are widely used as reinforcement in houses [117]. Hu et al. [118] explored innovative low carbon Engineered Cementitious Composites (ECC) incorporating sisal fibers and waste polyethylene fibers derived from discarded fishing nets. Using sisal fibers improved CO_2_ diffusion and uptake during carbonation curing, increasing compressive strength and tensile performance while reducing CO_2_ emissions by 50% compared to traditional ECCs.

##### Algae

**Figure 6:**
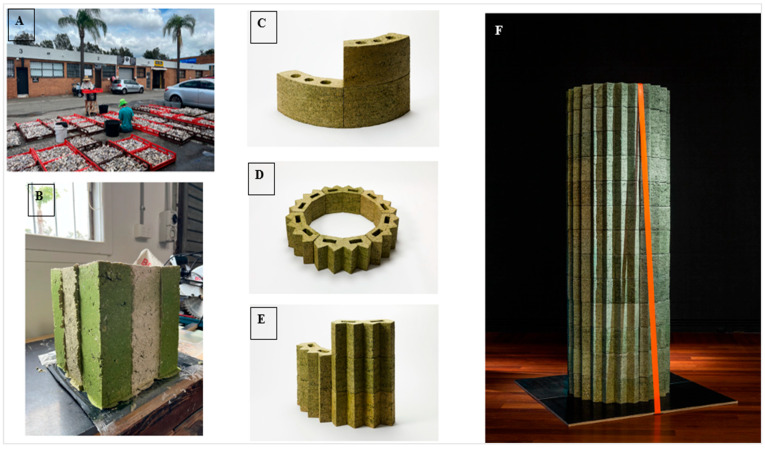
Developing and demonstrating seaweed-based biomasonry using macroalgae (Ulva) and waste oyster shell aggregate. (**A**) Cleaning, sorting, and sun-drying of oyster shells collected from oyster farms for use as a bio-aggregate. (**B**) Partially dried Ulva based cube prototype showing material shrinkage and layered composite texture. (**C**–**E**) Modular brick prototypes shaped in varying geometries: (**C**) curved bricks with hollow cores; (**D**) interlocking ring structure using zigzag-edge bricks; and (**E**) vertically fluted angular brick units for dry-stacking applications. (**F**) Full-scale demonstrator column (~2.6 m tall) constructed without mortar using 95 pressed bricks, exhibited at the Art Gallery of South Australia to showcase the architectural potential of seaweed biomaterials [23].

Algae are recognized as highly effective in capturing atmospheric CO_2_. After marine macroalgae (seaweed) is processed to produce high-value products, the leftover biomass can be repurposed for construction materials. Collaboration between designers and scientists has developed innovative marine macroalgae-based biomasonary products using a biorefinery approach [23]. The study optimized the material composition across four phases. Seaweed Ulva powder and carboxymethyl cellulose (CMC) emerged as the most effective binder, while crushed oyster shells were used as aggregates to improve stability and reduce shrinkage. A total of 95 bricks were used to construct a 2.6 m high column with a 900 mm diameter, showcased at an exhibition in Australia (Figure 6F). The research highlights the need for future work to compare the production embodied energy of seaweed bricks against commercially available clay bricks to assess their greenhouse gas (GHG) reduction potential. Additionally, algae-derived bio-oils have shown promise in enhancing asphalt pavement durability within the BioPave system [27]. Produced via hydrothermal liquefaction of algal biomass, these bio-oils improve thermal stability, UV resistance, and adhesive properties at optimal concentrations. For instance, bio-oils like Algae-1 (lake-harvested algal biomass) demonstrated high thermal stability, making them more suitable for applications in environments with high-temperature fluctuations. These findings emphasize algae’s versatility in decarbonizing the built environment sector, from biomasonry to infrastructure applications.

## 3. Direct CO_2_ Capturing Materials for the Built Environment

The built environment holds significant potential to shift from a passive to an active role in carbon mitigation. This section explores emerging materials and technologies capable of direct CO_2_ capture, suggesting their integration into construction materials. Although not yet widely applied, these innovations offer promising pathways for embedding carbon-capturing functions within the built environment.

**Figure 7:**
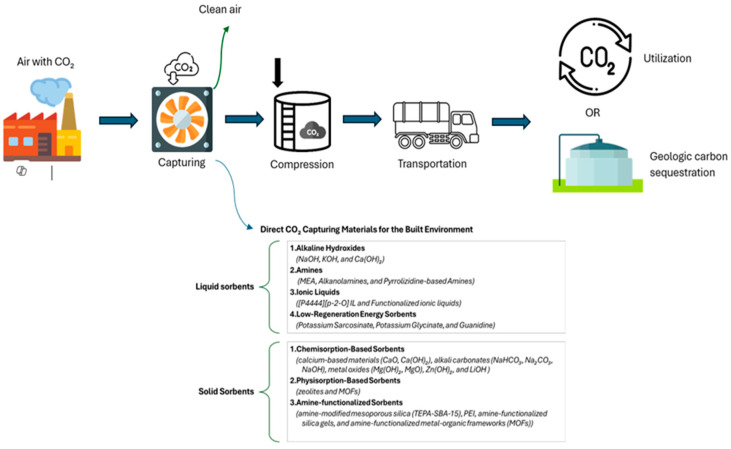
Schematic representation of the DAC technology and materials used for direct CO_2_ capture, including liquid and solid sorbents. These materials are being investigated for integration into construction materials to enable carbon-negative buildings.

Direct Air Capture (DAC) is an advanced technology designed to remove CO_2_ directly from the atmosphere, offering a scalable solution for climate change mitigation. Unlike conventional carbon capture methods focusing on industrial emissions, DAC can be deployed anywhere, making it highly adaptable for integration into the built environment. At the core of DAC technology are sorbent materials, specialized substances that selectively capture CO_2_ from the air. These materials capture CO_2_ from the air and release it during regeneration for reuse, storage, or industrial applications. Since CO_2_ is an acidic gas, sorbents are typically basic materials that efficiently bind with CO. Sorbents can be categorized into liquid sorbents, such as alkaline hydroxide solutions and amine-based solvents, and solid sorbents, including metal–organic frameworks (MOFs) and zeolites for example. The efficiency of DAC depends on the performance of these sorbents, which must offer high selectivity, fast CO_2_ uptake, low energy consumption for regeneration, and long-term stability. Figure 7 illustrates a comprehensive summary of all DAC sorbents found in this review. The following sections will explore each sorbent type in detail, beginning with liquid sorbents and then solid sorbents.

### 3.1. Liquid Sorbents

#### 3.1.1. Alkaline Hydroxides

**Figure 8:**
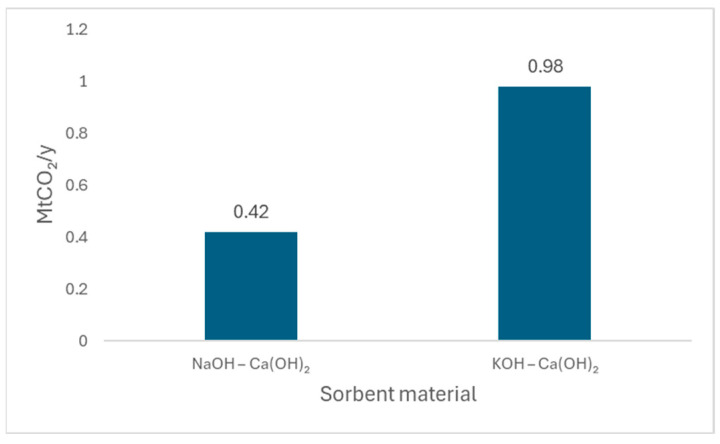
Comparison of annual CO_2_ capture capacities for two DAC systems using alkaline sorbents and calcium hydroxide. The NaOH–Ca(OH)_2_ system, based on the model by Baciocchi et al. [121], captures approximately 0.42 MtCO_2_ per year, while the KOH–Ca(OH)_2_ system, developed by Keith et al. [122], achieves a higher capture rate of 0.98 MtCO_2_ per year.

Alkaline hydroxides, including sodium hydroxide NaOH, potassium hydroxide KOH, and calcium hydroxide Ca(OH)_2_, are among the most widely studied liquid sorbents for Direct Air Capture (DAC) due to their strong chemical reactivity with CO_2_. These hydroxides absorb CO_2_ from the atmosphere and convert it into stable carbonate or bicarbonate compounds, effectively removing it from the air. NaOH and KOH are common alkaline sorbents that react efficiently with CO_2_ to form sodium carbonate (Na_2_CO_3_) and potassium carbonate (K_2_CO_3_) [119,120]. Figure 8 compares the CO_2_ capture capacity of two direct air capture (DAC) systems based on alkaline sorbents paired with calcium hydroxide recovery. The NaOH–Ca(OH)_2_ system, as modeled by Baciocchi et al. [121], achieves a capture rate of 0.42 MtCO_2_/year under modeled ambient conditions. In contrast, the KOH–Ca(OH)_2_ system, developed by Carbon Engineering and detailed by Keith et al. [122], demonstrates a significantly higher capture capacity of 0.98 MtCO_2_/year in a continuous, pilot-validated design. This improvement is attributed to optimized process integration and sorbent regeneration efficiency.

The primary limitation of NaOH and KOH-based systems is their high-temperature regeneration requirement (>900 °C), where the carbonate compounds must be decomposed in a calcination process to release pure CO_2_ and regenerate the hydroxide for reuse.

Alternative hydroxide regeneration cycles have been proposed to address this challenge, such as the NaOH–Na_2_O·3TiO_2_ cycle, significantly reducing energy consumption by nearly half [122].

Lackner implemented the Ca(OH)_2_ solution in DAC technology in 1999 [123]. While calcium hydroxide offers strong CO_2_ capture efficiency, its use in DAC is challenged by high energy requirements for calcination (179.2 kJ per mole of CO_2_), exceeding the theoretical minimum of 109.4 kJ per mole of CO_2_. Additionally, drying the precipitated CaCO_3_ further increases energy demand, making the process more resource-intensive. Another limitation is the low solubility of Ca(OH)_2_ in water, which restricts its concentration and overall CO_2_ uptake capacity.

#### 3.1.2. Amines

**Figure 9:**
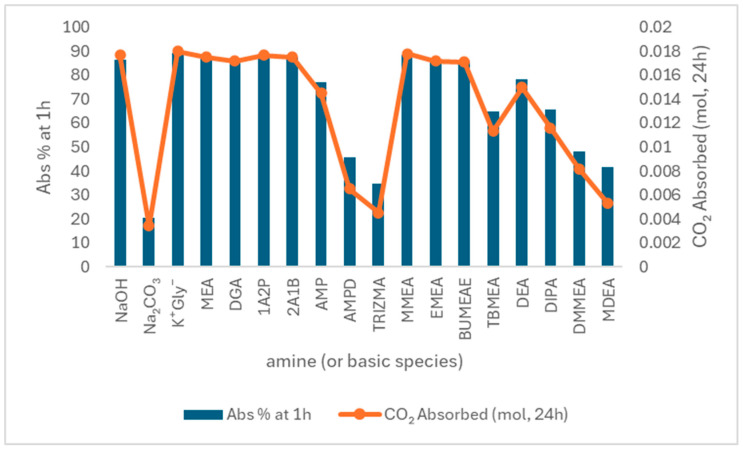
Comparison of CO_2_ Absorption Efficiency and Uptake by Alkanolamines Under Direct Air Capture Conditions (0.044% CO_2_ in Air) [127].

Amines, particularly alkanolamines, are a widely studied class of sorbents for Direct Air Capture (DAC). Aqueous monoethanolamine (MEA) has long been the benchmark for CO_2_ capture at industrial point sources. Still, it has proven inefficient for DAC, as its absorption rate drops significantly at low CO_2_ concentrations (~400 ppm) [124]. Additionally, alkanolamines exhibit lower absorption rates than alkaline hydroxide solutions, undergo oxidative degradation when exposed to oxygen, and require high regeneration energy due to water absorption, making them less favorable in their traditional form [125,126]. However, a recent screening study of different alkanolamines under DAC conditions (0.044% CO_2_ in air) showed that several amines reached CO_2_ absorption levels comparable to NaOH after 24 h, with similar absorption rates observed within the first hour [127]. These amines offer the added benefit of potentially lower regeneration energy requirements compared to aqueous alkali hydroxides. As shown in Figure 9, MEA, 1A2P, and 2A1B exhibited both high initial absorption and total uptake, while others, like MDEA, performed significantly lower, highlighting structural and chemical differences in capture efficiency.

Further advancements have been made in pyrrolizidine-based amine sorbents, which demonstrated stable CO_2_ absorption at 400 ppm over a nine-day period with no signs of oxidative degradation. However, the optimal absorption temperature was not specified [128]. Additionally, researchers have explored incorporating hydrophobic phenyl groups into alkylamines to eliminate water absorption, a major limitation of conventional amine-based systems. It was found that OXDA, MXDA, and PXDA did not absorb any water while still maintaining effective CO_2_ capture under DAC conditions [129]. While amines offer potential benefits such as lower regeneration temperatures and selective CO_2_ binding, challenges related to oxidative degradation, sorbent longevity, and environmental concerns remain significant hurdles.

#### 3.1.3. Ionic Liquids

Ionic liquids (ILs) are a promising type of sorbent for Direct Air Capture (DAC) because of their low melting points, high stability, and ability to be customized for better CO_2_ absorption [130,131]. Unlike traditional amine-based sorbents, ILs have low volatility and require less energy for regeneration, making them attractive for CO_2_ removal [132]. For DAC applications, Chen et al. [133] developed a pyrene-based polymer with [P4444] [p-2-O] IL, achieving 98.8% CO_2_ selectivity and enabling CO_2_ release using visible light, reducing the need for heat-based regeneration. Additionally, functionalized ILs containing amines can interact with CO_2_ physically and chemically, improving absorption at low CO_2_ concentrations [134,135]. However, ILs face challenges such as high production costs and difficulties in scaling up.

#### 3.1.4. Low-Regeneration Energy Sorbents

To overcome the high energy demands of conventional DAC systems, researchers have explored low-regeneration energy sorbents that operate at significantly lower temperatures while maintaining effective CO_2_ capture and release cycles. Custelcean et al. [136] introduced a DAC system using aqueous amino acid salts (potassium sarcosinate and potassium glycinate), where CO_2_ was captured and later crystallized into hydrated meta-benzene-bis(aminoguanidine) (m-BBIG) carbonate salts at room temperature. The carbonate crystals were then mildly heated (60–120 °C) to release CO_2_, regenerating the solid m-BBIG sorbent. Unlike alkaline hydroxide systems that require ~900 °C for regeneration, this approach significantly reduces oxidative and thermal degradation and can utilize low-grade waste heat sources. Another promising low-temperature sorbent was developed by Seipp et al. [137], utilizing an aqueous guanidine-based system for DAC. CO_2_ binds to guanidine, forming carbonate salts stabilized by weak guanidinium–hydrogen bonding, allowing for easy separation via filtration, eliminating the need for energy-intensive evaporation. The sorbent is regenerated at 80–120 °C, making it a more energy-efficient alternative to traditional amine or hydroxide-based DAC systems. The high energy demand for regeneration remains a significant challenge for liquid sorbents, making solid sorbents a more appealing option for Direct Air Capture (DAC). Unlike liquid sorbents, solid sorbents can be regenerated at lower temperatures (below 400 °C), reducing energy consumption. A detailed comparison of all liquid sorbents can be found in Table 4.

### 3.2. Solid Sorbents

#### 3.2.1. Chemisorption-Based Sorbents

Chemisorption sorbents are materials that capture CO_2_ through strong chemical bonds, typically offering high selectivity and capacity. Inorganic chemisorption sorbents, such as calcium-based materials (CaO, Ca(OH)_2_), alkali carbonates (NaHCO_3_, Na_2_CO_3_, NaOH), and metal oxides (Mg(OH)_2_, MgO), have demonstrated high CO_2_ adsorption capacities. However, NaHCO_3_ (Sodium Bicarbonate), Na_2_CO_3_ (Sodium Carbonate), and NaOH (Sodium Hydroxide) materials face challenges such as low adsorption rates and high regeneration temperatures (over 927 °C) [138], making them less practical for DAC applications. Additionally, calcium-based sorbents have shown higher adsorption rates than other inorganic sorbents but require elevated temperatures (above 400 °C) for optimal performance.

Although many face performance or scalability challenges, several metal hydroxides and oxides have been evaluated for CO_2_ capture from ambient air. Magnesium-based sorbents such as Mg(OH)_2_ and MgO exhibit slow adsorption kinetics, limiting their effectiveness in DAC applications [139]. Zn(OH)_2_ and LiOH have also been investigated, with Zn(OH)_2_ showing promising adsorption rates but failing to regenerate efficiently [140,141]. At the same time, LiOH has been used in submarine applications but is not regenerable. AgOH has shown low regeneration energy requirements, but its high cost makes it impractical for large-scale deployment [142].

#### 3.2.2. Physisorption-Based Sorbents

**Figure 10:**
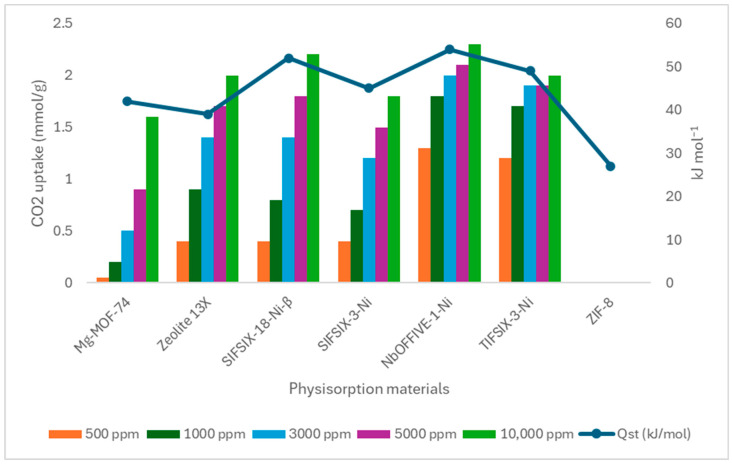
Comparative CO_2_ uptake and isosteric heat of adsorption for physisorbents at 298 K [145].

Physisorption sorbents are materials that capture CO_2_ through weak, reversible physical interactions, primarily van der Waals forces, rather than forming chemical bonds. Inorganic physisorption sorbents, such as zeolites (e.g., Faujasite X, Y, Zeolite 13X, K-LSX, Li-LSX, Na-LSX) and metal–organic frameworks (MOFs) (e.g., Mg-MOF-74, HKUST-1, SIFSIX-3-Ni), rely on these physical adsorption mechanisms. Zeolites, crystalline aluminosilicates, have high surface areas and tunable pore structures that enable selective CO_2_ adsorption. However, their performance declines in humid environments due to the competition between water vapor and CO_2_ for adsorption sites, which reduces CO_2_ selectivity [143,144]. Similarly, MOFs, highly porous hybrid materials composed of metal ions coordinated with organic linkers, have been extensively studied for DAC applications due to their tunable pore sizes and high CO_2_ selectivity. While MOFs exhibit promising selectivity, they also face challenges related to humidity, as water adsorption can block CO_2_ uptake. Compared to chemisorption materials, MOFs typically exhibit lower CO_2_ adsorption capacities [143,145]. Figure 10 presents the CO_2_ uptake capacities of various physisorbent materials at different concentrations (500–10,000 ppm) under DAC-relevant conditions (298 K). The ultramicroporous material SIFSIX-18-Ni-β, introduced by Mukherjee et al. [145], demonstrates a unique combination of moderate CO_2_ uptake (0.4 mmol/g at 500 ppm), high isosteric heat of adsorption (52 kJ/mol), fast kinetics, and low water affinity.

#### 3.2.3. Amine-Functionalized Sorbents

Researchers have developed amine-functionalized sorbents, which incorporate amines into porous solid supports to enhance CO_2_ selectivity and capture capacity. These materials work via chemisorption, where CO_2_ reacts with amine groups to form carbamates or bicarbonates, enabling potent and selective adsorption even at low CO_2_ concentrations. TEPA-SBA-15, an amine-modified mesoporous silica, demonstrates significant performance in humid conditions, which is crucial for effective CO_2_ capture, as it overcomes the limitations faced by many physisorbents [145]. Additionally, poly(ethyleneimine) (PEI) impregnated sorbents have shown versatility, achieving maximum adsorption capacities of 4.1 mmol/g in flue gas and 2.6 mmol/g in direct air capture conditions, indicating their adaptability to varying environments [146]. Furthermore, amine-functionalized silica gels have been developed, with wet-grafting conditions yielding a CO_2_ capture capacity of 1.098 mmol/g, significantly higher than that achieved under dry conditions [147]. Moreover, amine-functionalized metal–organic frameworks (MOFs) offer enhanced CO_2_ selectivity and capture capacity, although pore-blocking effects remain a challenge that limits adsorption efficiency [148].

## 4. Future Directions

The built environment is recognized as a major contributor to global CO_2_ emissions, accounting for a substantial share of energy consumption and material-related emissions. However, it also holds significant potential to act as a carbon sink by leveraging innovative materials and technologies, circular economic approaches, policy implementation, and standards strategies.

### 4.1. Innovative Materials and Technologies

Numerous materials have demonstrated significant carbon capture, storage, and reduction potential within the built environment. Bio-based products, industrial by-products, and construction and demolition wastes have been extensively explored for their ability to sequester CO_2_ while enhancing material sustainability. However, limited research has focused on directly integrating CO_2_-absorbing agents, such as zeolites, into building materials to enhance their carbon sequestration capacity. Zeolites, with their high surface area and adsorption properties, could be crucial in capturing atmospheric CO_2_ when incorporated into cementitious composites [149], yet their full potential remains underexplored. The study investigated the effects of adding LTA 5A and LTA 4A zeolites (3–12 wt%) to cement mortars. While both zeolites improved early strength, LTA 5A maintained its strength over time, whereas LTA 4A experienced strength loss due to increased porosity. CO_2_-impregnated zeolites significantly enhanced compressive strength and hydration, with CaCO_3_ content increasing by 5.3 wt% for LTA 5A and 4.8 wt% for LTA 4A. LTA 5A demonstrated superior CO_2_ absorption and mechanical performance, making it a promising option for in situ carbonation in cement-based materials.

Another promising yet underutilized technology is additive manufacturing (AM), which enables precise material optimization, reduced waste generation, and enhanced structural efficiency. While AM has been increasingly applied in sustainable construction, few studies have investigated its integration with carbon-capturing materials. Future research should explore the synergistic potential of additive manufacturing and CO_2_-capturing materials, focusing on material formulation, structural integrity, and large-scale feasibility.

### 4.2. Synergic Carbon Storage and Circular Economy Approach

**Figure 11:**
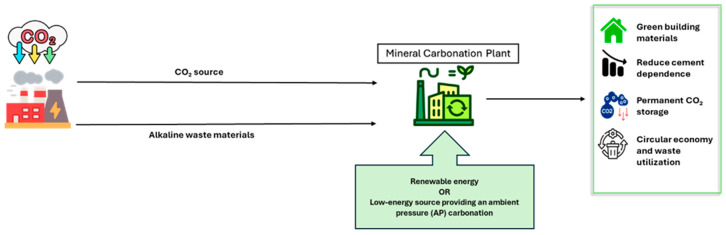
Schematic representation of a mineral carbonation process integrating industrial CO_2_ emissions and alkaline waste materials. CO_2_ captured from emission sources and combined with alkaline waste in a mineral carbonation plant enables the production of sustainable construction materials. Powered by renewable or low-energy sources (e.g., ambient pressure carbonation), the process facilitates green building applications, reduces cement dependency, ensures permanent CO_2_ storage, and promotes circular economy and waste valorization.

Figure 11 illustrates the closed-loop process of mineral carbonation, where industrial exhaust gases are used as a CO_2_ source that reacts with alkaline materials to form stable carbonates. This process prevents landfill disposal and transforms industrial by-products into valuable construction materials, supporting both carbon storage and material circularity. As previously discussed, alkaline materials such as industrial waste and construction and demolition waste (CDW) are particularly suitable for this process due to their high content of calcium and magnesium oxides, which readily react with CO_2_. Carbonating these waste materials enables long-term carbon sequestration while promoting their reuse in cementitious applications, contributing directly to carbon reduction strategies in the built environment. The integration of ambient pressure (AP) carbonation further enhances this cycle by providing a low-energy, scalable solution for CO_2_ sequestration [57,70]. Unlike traditional high-pressure methods, AP carbonation allows for directly treating industrial waste on-site, eliminating the need for energy-intensive gas compression. This approach is particularly advantageous when implemented at industrial facilities co-located with alkaline waste sources, as it ensures greater carbon capture efficiency and sustainable material production.

### 4.3. Policy Implementation and Standards

Despite advancements in carbon-sequestering materials, there remains a lack of standardized policies and regulations governing their application in construction. Clear guidelines, certification frameworks, and regulatory incentives are needed to ensure their scalability and industrial adoption. Future policies should integrate carbon sequestration requirements into building codes and sustainability certification. Additionally, future research should focus on the large-scale feasibility of these materials, including cost analysis, supply chain optimization, and industrial integration.

### 4.4. Life Cycle Assessments and Economic Analysis Integration to Practice

A key direction for advancing CO_2_-capturing, reduction, and storage technologies is the integration of comprehensive life-cycle assessment (LCA) and economic analysis. Li et al. [67] showed through LCA that aqueous carbonation of steel slag has a GWP of 96.2–24.9 kg CO_2_-eq/t, offsetting additional grinding emissions and achieving the lowest impact. This optimal size also produced a positive economic return of 16.5 CNY/t, highlighting the value of LCA in identifying sustainable process conditions. Kravchenko et al. [76] showed that the environmental benefits of carbonation are highly sensitive to the electricity mix; shifting to a higher-carbon grid increased curing and compaction emissions by 5.53 and 14.08 kg CO_2_-eq/t, respectively, turning an otherwise carbon-negative process into a net-positive GWP of 12.57 kg CO_2_-eq/t. Xian et al. [57] further demonstrated that ambient-pressure carbonation offers substantial environmental and economic advantages, avoiding 224.7 kg of CO_2_ emissions per ton of steel-slag concrete and reducing production costs from $ 206.3/ton (OPC pipes) to $ 39.2/ton, making large-scale deployment more viable near industrial CO_2_ sources. Moreover, Zhang et al. [82] confirmed the practical relevance of these benefits through a road-widening project in Suzhou, where the replacement of conventional foamed concrete with MgO-based CO_2_-foamed concrete reduced embodied emissions from 1000 to 470 tons, while enabling long-term CO_2_ sequestration, resulting in a total reduction of 530 tons for the project. Future research should prioritize Life Cycle Assessments and Techno-Economic Analysis (TEA) to compare different carbonation technologies and incorporate industrial case studies that better connect laboratory findings with real-world practice.

## 5. Conclusions

The built environment (BE) is vital in reducing global carbon emissions; the sector’s high carbon intensity emphasizes the need for climate-resilient construction practices. This review examined current carbon capture, storage, and reduction strategies within the built environment, focusing on material levels. Carbon capture approaches such as the photosynthesis-based method, concrete carbonation, and biomineralization show potential for embedding CO_2_ absorption directly into construction processes. Photosynthesis-based materials utilize the natural carbon fixation abilities of plants, agricultural residues, and algae to capture atmospheric CO_2_ before being processed into construction components. While these materials have the potential to be carbon-negative and renewable, challenges still exist concerning their durability and integration into structural systems. Concrete carbonation involves the reaction of CO_2_ with calcium hydroxide in cementitious materials, forming calcium carbonate that densifies the matrix and may enhance durability. Although it provides permanent carbon storage, the process is slow, particularly under ambient CO_2_ concentrations. Moreover, the high energy demand and emissions associated with OPC production challenge its overall sustainability. Biomineralization, mainly microbially induced carbonate precipitation (MICP), utilizes microorganisms to convert CO_2_ into stable mineral carbonates within construction materials. This method presents promising opportunities for CO_2_ sequestration and self-healing capabilities; however, it remains at the experimental stage, with further research needed to address scalability, cost-effectiveness, and long-term performance.

Mineral carbonation and carbonation curing are two well-established methods for carbon storage that hold significant promise for use in the construction sector. Mineral carbonation involves the reaction of carbon dioxide (CO_2_) with alkaline materials, such as industrial waste rich in calcium or magnesium, to form stable carbonates like calcium carbonate. This process enables long-term and permanent storage of CO_2_. In contrast, carbonation curing occurs during the early stages of setting for binders or concrete. In this process, CO_2_ is introduced under controlled conditions to accelerate the strength gain of the material while sequestering carbon. This method enhances early-age strength and durability and reduces the curing time of concrete. Both approaches have significant advantages but typically rely on controlled environments and access to concentrated CO_2_ streams. This reliance introduces challenges regarding scalability and cost, especially for large-scale or decentralized applications. However, recent studies have shown that ambient pressure (AP) carbonation is feasible. This method directly treats industrial waste and construction and demolition waste, eliminating the need for energy-intensive CO_2_ compression systems. This advancement greatly improves the practicality and energy efficiency of carbonation-based strategies in real-world construction settings.

Carbon reduction strategies that focus on replacing carbon-intensive materials with sustainable alternatives present significant opportunities for lowering emissions in the built environment. Industrial by-products such as fly ash, steel slag, red mud, and ground granulated blast furnace slag (GGBFS) are commonly used as supplementary cementitious materials (SCMs) and alkali-activated binders. These materials help reduce the demand for clinker and contribute to CO_2_ sequestration through carbonation. Additionally, bio-based materials sourced from renewable resources like plants, algae, and agricultural residues have been utilized in various applications, including insulation, wall systems, bricks, panels, and structural and non-structural components. This showcases their versatility and environmental benefits.

The study also examined the current materials used for direct CO_2_ capture and found that emerging Direct Air Capture (DAC) materials present a promising opportunity for integrating active CO_2_ sequestration into the built environment. Materials like zeolites and other porous composites demonstrate a high capacity for CO_2_ adsorption and show potential for multifunctional use in construction materials. However, their practical application is still limited due to scalability and long-term performance challenges, highlighting the need for further research and development in this area.

Building on previous advancements, future research should prioritize integrating carbon-sequestering innovations into mainstream construction practices. Promising strategies involve incorporating CO_2_-adsorbing agents into construction materials, enhancing passive carbonation and maintaining structural performance, ultimately allowing buildings to function as carbon sinks. Additionally, the synergistic application of additive manufacturing with low-carbon materials offers an opportunity to optimize resource efficiency, minimize waste, and improve carbon capture. The advancement of ambient pressure mineral carbonation also offers a scalable, low-energy solution for CO_2_ storage, particularly when located near industrial waste sources. Finally, for these innovations to be widely adopted, it will be essential to develop standardized policies, certification frameworks, and life-cycle assessment tools that support the industrial deployment of carbon-negative materials.

In conclusion, changing the built environment from a carbon emitter to a carbon sink is achievable and crucial for reaching global net-zero targets. Integrating innovative materials, principles of a circular economy, and supportive policies will be vital in transforming the future of construction. By utilizing carbon capture, storage, and reduction strategies, the built environment can become active in climate mitigation, paving the way for a sustainable, carbon-neutral future.

## Figures and Tables

**Table 1:** Materials captured CO_2_ by photosynthesis mechanism.

Material Mix	Biomass Source	Production Process	Application in the Built Environment	Ref.
Wet Ulva Ohnoi paste, a by-product derived from a biorefinery process Carboxymethyl Cellulose (CMC) as binder Crushed oyster shells as aggregates	Residual biomass from seaweed cultivation	Biorefinery	Bricks	[23]
OPC Grade 43 Biochar replaced cement at 3%, 5%, and 10% by weight Water-to-cement (W/C) ratio: 0.5	Rice husk	Pyrolysis at 500 °C for 2 h in a muffle furnace	Cementitious paste	[25]
Hemp hurds and a bio-based binder	Hemp hurds from industrial hemp (*Cannabis sativa* L.)	Decortication: Hemp stalks are crushed to separate hurds (used in the boards) from fibers and dust Thermocompression: The mixture is compressed using a heated hydraulic press to produce 1 m^2^ boards	Hemp-based boards	[21]
Lignoboost lignin (LB), Lignoforce lignin (LF), alkali lignin, and hydrolysis lignin Grade 90 sand as aggregates Dimethyl sulfoxide (DMSO) as solvent	Byproducts from: Paper and pulp industry (kraft and alkaline pulping) Bioethanol and cellulose industries	Lignin types sourced via sulfate, alkaline, and hydrolysis processes	Ligno Block Non-structural building elements	[26]
Biochar core Aggregate shell composed of CEM I 52.5 cement (OPC) and ground granulated blast furnace slag (GGBS) Water-to-binder (W/B) ratio: 0.21	Corncob waste	Pyrolysis of corncob waste at 500 °C for 2 h	Aggregates	[20]
Type I OPC Fine aggregates (mean size 0.86 mm) Biochar blend with coal: 0%, 5%, 10%, and 15% by weight of cement	Pure rice husk	Rice husk blended with coal in a 3:1 ratio Pyrolyzed at 500 °C	Filler	[19]
Bitumen, bio-oil (3% and 6%), and glass beds	Algae-1: Lake-harvested algal biomass Algae-2: Lake-harvested algae blended with Engineered BioSlurry (EBS) Algae-3: Algal biomass blended with wood powder Algae-4: Wild-type seaweed (e.g., Ulva species) Algae-5: Post-extraction Haematococcus pluvialis biomass wastewater sludge	Hydrothermal liquefaction Reactor temperature: 320–340 °C Pump pressure: 2800–2900 psig	Biopave	[27]
Biochar and PET blended in three ratios: 90:10, 70:30, and 50:50	Wastewater sludge	Wastewater sludge pyrolyzed at 700 °C to produce biochar Biochar mixed with PET pellets to form a composite	Tiles	[28]
Cellulose fibrils mixed with Silica aerogel precursor	Wheat straw	Alkaline Treatment (NaOH-Based)	Insulation panels	[29]
Cement, fine aggregates, and superplasticizers, Nanobiochar (0.00%, 0.04%, 0.06%, 0.08%, 0.12%, and 0.15%) W/B is 0.47	Apricot kernel shell	Pyrolysis at 500 °C	Filler	[30]
Hemp Shives, Binder composed of: 75% hydrated lime, 15% Natural Hydraulic Lime, 10% Metakaolin, and water	Hemp stalks (shiv/hurds)	Hydrated lime produced by extracting, crushing, and burning limestone at 900–1200 °C	Hemp-lime concrete wall	[31]
Cement, sand, SCBA replaced sand at levels of 50% and 75%, Zeolite, and polypropylene fibers Water-to-cement (W/C) ratios: 0.38 and 0.51	Residue from sugarcane bagasse combustion	Sugarcane bagasse ash (SCBA) dried at 65 °C for 12 h Sieved using a No. 20 sieve to remove coarse particles	Pavement repair mortars	[32]
Steel–GluBam hybrid truss consisting of: Upper and diagonal web poles: GluBam Lower chord pole: Steel pipe	Bamboo grown in China		Steel-GluBam hybrid truss	[33]
OPC or MOSC binders Supplementary cementitious materials (SCMs): fly ash, GGBS Biochar (50–70%)	Wood waste	Wood waste pyrolyzed at 700 °C Biochar pre-soaked to enhance cement hydration	Aggregates in cement-bonded biochar particleboards	[34]
Case 1: Masonry-concrete straw bale rural house Case 2: Wood frame straw bale rural house Case 3: Lightweight steel straw bale rural house Case 4: Steel frame straw bale rural house Case 5: Reference ordinary rural house.	Wheat straw		Straw bale rural house	[35]
SSIA and OPC mixed in a 1:1 ratio Biochar added at 1–5% Water-to-binder (W/B) ratio: 0.45	Peanut shells	Biochar produced by pyrolyzing peanut shells at 500 °C and 700 °C	Lightweight concrete	[36]
OPC, biochar with dosages of (0, 1%, 3%, 5%), river sand, and superplasticizer Water-to-cement (W/C) ratio: 0.4	Corn straw	Corn straw pyrolyzed in a vacuum furnace at 300 °C, 400 °C, 500 °C, and 550 °C for 1.5 h	Filler	[37]
OPC, quartz sand, and biochar replacing cement at 2%, 5%, 10%, 15%, and 20%	Wood chip	Pyrolysis	Filler	[38]
FBC fly ashes and polycarboxylate-based superplasticizer	Combustion of peat and wood	Biomass fly ash collected from fluidized bed combustion (FBC)	Tiles	[39]
Treated hemp shive, bio-based binder, timber frame, and Steel fastening	Hemp shiv	Shiv treated with Sol–Gel coating Mixed with bio-based binder and water to enhance durability and reduce water absorption	Hempcrete wall	[40]

**Table 2:** Different microbial approaches to form CaCO_3_.

Bacteria Used	Metabolization Mechanism	Carbonation Condition	CO_2_ Uptake/Reduction	Application	Ref.
Genetically modified *Bacillus subtilis*. These CA enzymes were taken from *Bacillus megaterium*	Carbonic anhydrase	Temperature of 30 °C, concentration of CO_2_ at 3800, and duration of 72 h	Reduction in CO_2_ levels (from 3800 ppm to 820 ppm)	Cementitious materials	[51]
*Bacterium* SAML2018	CA and urease	Temperature of 25 °C, concentration of CO_2_ at 3%, and duration of 24 h	72.96% carbonation degree after 28 days for Ureolysis solution medium 44% carbonation degree after 28 days for the CO_2_ hydration solution medium	Cementitious materials and concrete cracking repair	[50]
*Streptomyces microflavus* *Paenibacillus mucilaginosus*	CA	Curing in the carbonation box with a 10% carbon dioxide concentration. Treatment was repeated 1, 3, 5, and 7 times, and each treatment was 24 h		Dust Control in sand	[49]
*Synechococcus* PCC8806 *Synechocystis* sp. PCC6803 *Synechococcus* sp. LS0519 *Synechococcus* sp. PCC8806	Oxygenic photosynthesis (by Cyanobacteria)	Atmospheric CO_2_		Concrete cracking repair	[52]

**Table 4:** Different Liquid Sorbent Types.

Liquid Sorbent Type	Examples	CO_2_ Capture Mechanism	Regeneration Temperature	Advantages	Limitations	Ref.
Alkaline Hydroxides	NaOH, KOH, and Ca(OH)_2_	Forms stable carbonates (Na_2_CO_3_, K_2_CO_3_, CaCO_3_)	>900 °C	High CO_2_ absorption capacity	Very high regeneration energy, Ca(OH)_2_ has low solubility	[119,120,123]
Amines	MEA, Alkanolamines, and Pyrrolizidine-based Amines	Binds CO_2_ via acid-base reactions		Some amines have lower regeneration temperatures than hydroxides	Aqueous Alkanolamines have lower absorption rates compared to alkaline liquid sorbents	[124,127,128,129]
Ionic Liquids	[P4444][p-2-O] IL and Functionalized ILs	Physical and chemical interactions		Have low melting points and can be tailored for specific properties Offer lower energy regeneration and low volatility	High production cost and scalability challenges	[133,134]
Low-Regeneration Energy Sorbents	Potassium Sarcosinate, Potassium Glycinate, and Guanidine	Crystallization of carbonate salts (m-BBIG, guanidine)	60–120 °C	Low regeneration temperature	The amino acid salts are susceptible to oxidative and thermal degradation. In guanidine-based systems, the formation of carbonated crystals can complicate the regeneration process	[136,137]

## Data Availability

No new data were created or analyzed in this study. Data sharing is not applicable.
